# Integrating multimodal clinical data with a large model for prostate cancer diagnosis

**DOI:** 10.1038/s41746-026-02670-x

**Published:** 2026-04-25

**Authors:** Chengbang Wang, Yuan Tian, Shaojie Yin, Xuhong Zhang, Xuedong Wei, Lingfeng Wu, Zhengdong Zhou, Guijian Pang, Yan Wang, Wangjian Wu, Shukai Zhao, Ziwei Wang, Jiangnan Xu, Hao He, Minglun Li, Zhankui Jia, Xu Gao, Fubo Wang, Guangtao Zhai, Bin Xu

**Affiliations:** 1 Department of Urology, Shanghai Ninth People’s Hospital, Shanghai Jiao Tong University School of Medicine, Shanghai, China; 2 Shanghai Artificial Intelligence Laboratory, Shanghai, China; 3 Institute of Image Communication and Network Engineering, Shanghai Jiao Tong University, Shanghai, China; 4 Department of Urology, The First Affiliated Hospital of Soochow University, Suzhou, China; 5 Department of Urology, The Affiliated Hospital of Jiaxing University, Jiaxing, China; 6 Department of Urology, Yancheng First Hospital, Affiliated Hospital of Nanjing University Medical School, The First people’s Hospital of Yancheng, Jiangsu, China; 7 Department of Urology, The First People’s Hospital of Yulin (the Sixth Affiliated Hospital of Guangxi Medical University), Yulin, Guangxi China; 8 Department of Urology, Changhai Hospital, Second Military Medical University (Naval Medical University), Shanghai, China; 9 Department of Urology, The First Affiliated Hospital of Zhengzhou University, Zhengzhou, China; 10 Department of Radiation Oncology, Hospital Lüneburg, Lüneburg, Germany; 11 Center for Genomic and Personalized Medicine, Guangxi key Laboratory for Genomic and Personalized Medicine, University Engineering Research Center of Digital Medicine and Healthcare, School of Life Science, Guangxi Medical University, Nanning, Guangxi China

**Keywords:** Cancer, Computational biology and bioinformatics, Medical research, Oncology

## Abstract

Accurate prostate cancer (PCa) diagnosis remains difficult because of tumor heterogeneity and the challenge of integrating multimodal clinical information. We developed Prost-LM, a multimodal large language model that jointly embeds MRI-derived features, numerical PSA values, and free-text clinical reports into a unified semantic space to enable deep cross-modal reasoning. Trained and validated on a large multi-center cohort of 3940 patients, Prost-LM achieved strong diagnostic performance, with an internal validation AUC of 0.954 for distinguishing PCa from benign conditions, outperforming MRI-only models (AUC = 0.868, *P* < 0.001). For detecting clinically significant PCa (Gleason score ≥ 7), Prost-LM reached an AUC of 0.955. Additionally, the model provides interpretable diagnostic decisions to support clinical verification. These results suggest Prost-LM can improve automated PCa diagnosis and support precision oncology through multimodal AI.

## Introduction

Prostate cancer (PCa) is the second most commonly diagnosed malignancy among men worldwide, and its incidence has steadily increased in recent years^1^. Owing to its insidious onset and lack of overt early symptoms, the proportion of late-stage diagnoses has risen significantly from 3.9% to 8.2%^2^, thereby complicating treatment and adversely affecting patient prognosis. Accurate and non-invasive diagnostic approach among PCa, benign prostatic hyperplasia (BPH), and other benign prostatic conditions is therefore critical for guiding clinical decision-making, minimizing unnecessary invasive interventions, and optimizing therapeutic outcomes^3^.

Magnetic resonance imaging (MRI) has emerged as a pivotal tool in the diagnostic workup of prostatic disease. For standardized interpretation and reporting, clinical practice widely relies on the Prostate Imaging Reporting and Data System version 2 (PI-RADS v2)^4^. However, the diagnostic performance of MRI when interpreted under the PI-RADS v2 guidelines is still considered unsatisfactory. For instance, a meta-analysis reported a sensitivity of 0.89 and a specificity of 0.73 for PCa detection^5^, limitations often attributed to a grading strategy that is overly simple and lacks flexibility^6–9^.

The evolution of artificial intelligence (AI) in PCa diagnosis began with conventional machine learning approaches. Early studies utilized statistical models such as Logistic Regression (LR)^10,11^, Support Vector Machines (SVM)^12^, and Random Forests^13^, integrating structured clinical variables (e.g., Prostate-Specific Antigen (PSA)^14^, age) with handcrafted radiomic features from MRI^15^. Contemporary risk calculators further refined these using routine clinical parameters without heavy reliance on imaging^16^. While interpretable and effective in reducing unnecessary biopsies, these methods depended on manual feature engineering and struggled with raw high-dimensional imaging or unstructured data.

Building upon this, deep learning (DL) has emerged as a promising alternative with automated feature learning^17^. Most DL methods focus on MRI analysis, progressing from radiomics^15,18^ to end-to-end models^17,19,20^. Integrated approaches combine DL imaging features with PI-RADS and clinical variables for better csPCa detection on biparametric MRI^21^. Recent multimodal DL models further improve risk stratification and reduce unnecessary biopsies by fusing clinical and MRI data^22^. Other efforts employ neural networks or ML to integrate MRI (including PI-RADS), clinical, and hematologic data for prebiopsy risk prediction^23,24^, with large multicenter studies showing improved csPCa detection via multimodal fusion^25^. However, many still use simplistic fusion strategies (e.g., logistic regression after concatenation), failing to capture complex interactions between heterogeneous sources.

Concurrently, recent advances in large language models (LLMs)^26^ offer an emerging solution that directly addresses these limitations. Unlike prior methods that rely on simplistic fusion techniques, LLMs leverage their massive scale and sophisticated architectures to perform deep fusion. They can project highly heterogeneous data, such as MRI images, numerical PSA values, and textual patient reports, into a unified, high-dimensional semantic space^27^. Within this space, LLMs can better model the complex, non-linear interactions between these modalities, a capability that eluded previous multi-modal AI architectures^28–30^. This capability holds the promise of fully exploiting the synergistic value of all available clinical data for a more accurate and robust PCa diagnosis.

**Fig. 1:**
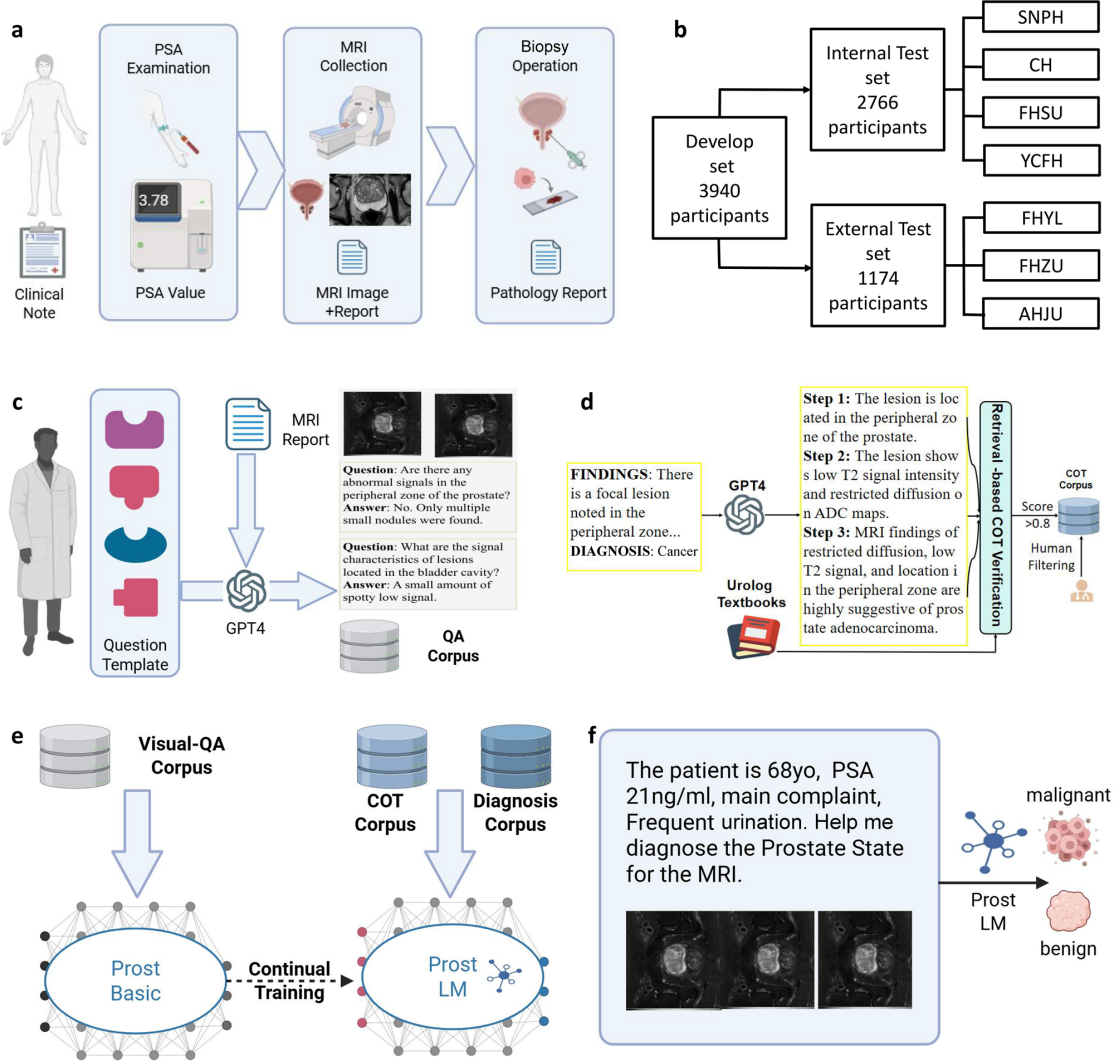
Framework of Prost-LM: A multimodal LLM for explainable prostate cancer diagnosis. **a** Clinical workflow. Patients first undergo biochemical testing (measuring tPSA and fPSA levels), followed by MRI with standardized PI-RADS assessment. All cases receive histopathological biopsy confirmation with Gleason grading as the diagnostic gold standard. **b** Multicenter validation design. The model was developed using data from four centers (training/internal validation), with independent external validation performed across three additional centers to ensure generalizability. **c** Visual-QA corpus construction. We developed a diagnostic question-answering dataset by: (1) processing paired MRI images and radiology reports using GPT4, and (2) incorporating expert-curated template Q& A pairs from experienced radiologists to guide natural language generation. **d** Chain-of-thought (CoT) corpus generation. The CoT dataset was created by: (1) extracting domain knowledge from urology textbooks, (2) using GPT4 to generate reasoning chains combining radiology reports with medical knowledge, and (3) physician-led quality control through manual filtering. **e** Progressive training paradigm. The model development occurred in two stages: (1) ProstQA - trained on Visual-QA data for MRI and report comprehension, and (2) Prost-LM - extended through continual learning on CoT and diagnostic corpora to integrate multimodal data (imaging, PSA values, clinical symptoms) and provide interpretable, stepwise diagnostic reasoning. **f** Inference pipeline. During deployment, clinical variables are structured via prompt templates while MRI data is processed in parallel, enabling Prost-LM to generate comprehensive diagnostic assessments with explanatory reasoning. Icons are from https://uxwing.com/and https://www.biorender.com/. Created with BioRender.com.

Here, we present Prost-LM, the first large multimodal model specifically engineered for PCa diagnosis (Fig. 1). Prost-LM is developed using a comprehensive cohort of 2,213 cases, each with complete clinical records. First, the model learns fundamental concepts of prostate pathology by ingesting a knowledge base of image-text pairs derived from medical textbooks. Subsequently, it undergoes extensive fine-tuning on a large-scale, multi-modal dataset tailored for diagnosis. This dataset consists of question-answering pairs, where GPT-4 was prompted with radiology reports and clinical data to generate realistic MRI-based questions and answers. This process, combined with diagnostic training data, enables Prost-LM to effectively correlate imaging findings with crucial clinical variables (e.g., PSA) and patient notes, ultimately supporting its diagnostic task.

Validated across an internal cohort and three independent external cohorts, Prost-LM demonstrates strong and broadly generalizable diagnostic performance. In distinguishing PCa from benign prostatic disease, it achieved an AUC of 0.954 (95% CI 0.941–0.966) in the internal cohort and consistently outperformed baseline models across external centers, with AUCs up to 0.800. For clinically significant PCa (csPCa), Prost-LM reached an AUC of 0.955 (95% CI 0.942–0.968) internally and maintained performance advantage in all external cohorts, achieving AUCs as high as 0.811. Decision curve analysis (DCA) further confirmed that the Prost-LM-guided strategy yields the highest net benefit in diverse clinical scenarios. Prost-LM has the potential to enhance the diagnostic workflow, offering a more precise, robust, and standardized approach to PCa management that can alleviate the burden on both patients and healthcare systems.

## Results

### Cohort building

**Fig. 2:**
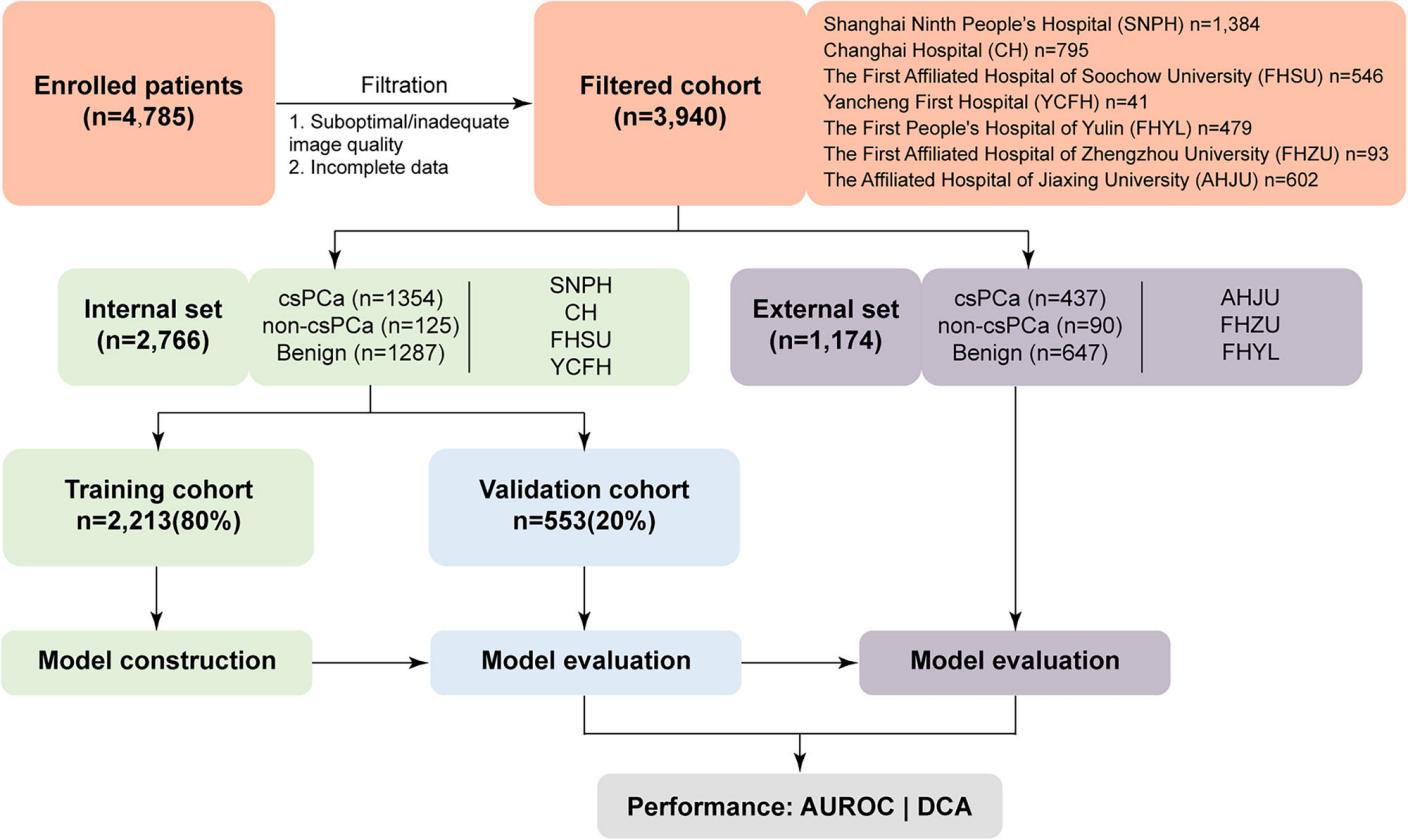
Study design. AUROC area under curve-receiver operating characteristic curve, DCA decision curve analysis.

This retrospective diagnostic study enrolled 4785 male patients (Fig. 2) aged 21–94 years who underwent MRI and had definitive diagnoses between January 2018 and December 2024 at seven clinical centers: Shanghai Ninth People’s Hospital (SNPH), Changhai Hospital (CH), The First Affiliated Hospital of Soochow University (FHSU), The Affiliated Hospital of Jiaxing University (AHJU), The First Affiliated Hospital of Zhengzhou University (FHZU), YanCheng First Hospital (YCFH), and The First People’s Hospital of Yulin (FHYL). Tumor characteristics and Gleason scores were extracted from pathological reports by board-certified pathologists. More details of patient characteristics are in the Supplementary Table 1.

The cohort was stratified for analysis as follows (Fig. 2): 80% of the data from SNPH, CH, FHSU, and YCFH were adopted as the training set. The remaining 20% were adopted as the internal validation set. The other three clinical centers, namely, FHYL, AHJU, and FHZU, are adopted as the external validation set.

### Prost-LM achieves improved PCa diagnosis performance

**Fig. 3:**
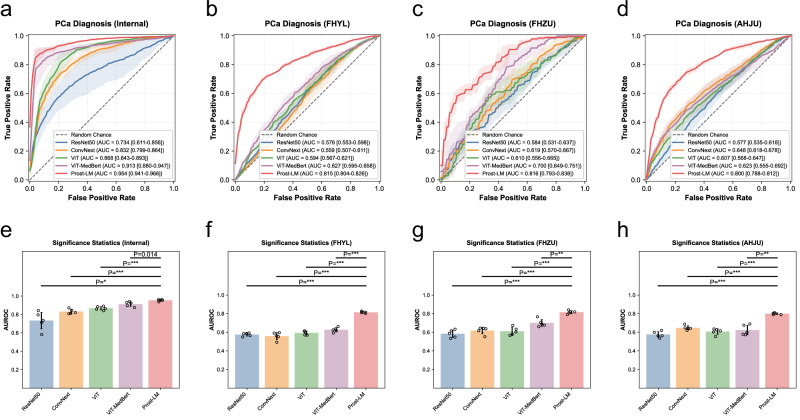
Performance evaluation in prostate cancer diagnosis. **a**–**d** Comparison of classification efficacy of different diagnostic methods in the internal and external sets. **e**–**h** Statistical significance (P-values) between Prost-LM and comparative approaches, with asterisks indicating significance levels: *P < 0.01, **P < 0.001, and ***P < 0.0001. All *P*-values were calculated using two-sided *t*-tests.

We first conducted a comprehensive evaluation on our internal validation cohort for overall PCa diagnosis. Our proposed Prost-LM achieved an AUC of 0.954 (95% CI: 0.941–0.966), which was statistically improved compared to all baseline methods, including the clinical multimodal baseline ViT-MedBert (AUC = 0.913; *P* = 0.014) and all vision-only models (*P* < 0.01 for all; Fig. 3a, e). To further assess the stability of Prost-LM for PCa diagnosis across different data stratifications, we implemented a 5-fold cross-validation scheme on the internal dataset (Supplementary Fig. 1a, c). In each fold, the cohort was restructured into Training (60%), Internal Validation (20%), and Internal Testing (20%) sets. Prost-LM achieved a mean AUC of 0.950 (95% CI: 0.938–0.963) across all 5 folds. Paired t-tests confirmed that the performance improvement of Prost-LM over the strongest baseline (ViT-MedBert) remained statistically significant (*P* < 0.05). To assess the model’s generalizability, we further validated it on three independent external cohorts (FHYL, FHZU, and AHJU). Prost-LM consistently maintained its performance advantage, achieving the highest AUC in all three cohorts and significantly outperforming all comparative models (all *P* < 0.05; Fig. 3b–d, f–h).

**Fig. 4:**
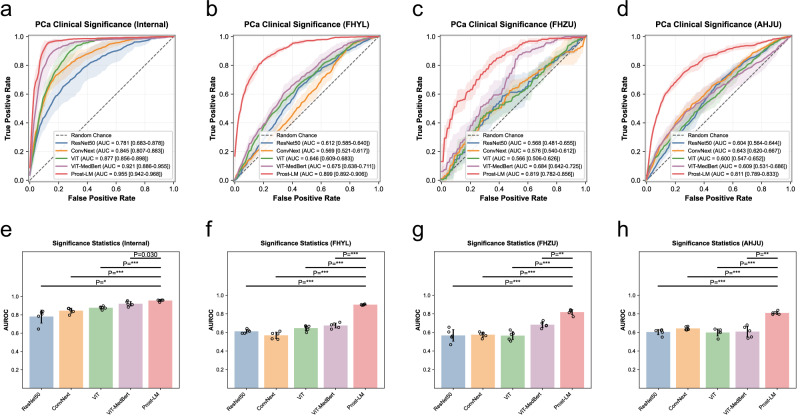
Performance evaluation in clinically significant prostate cancer diagnosis. **a–d** Comparison of classification efficacy of different diagnostic methods in the internal and external sets. **e–h** Statistical significance (*P*-values) between Prost-LM and comparative approaches, with asterisks indicating significance levels: *P < 0.01, **P < 0.001, and ***P < 0.0001. All *P*-values were calculated using two-sided *t*-tests.

Prost-LM demonstrated great performance gains in diagnosing csPCa (Gleason score ≥ 7), which is the primary target for clinical intervention. In the internal cohort, our model attained a strong AUC of 0.955 (95% CI: 0.942–0.968) for csPCa detection. This represents a significant improvement over the best-performing baseline, ViT-MedBert (AUC = 0.921; *P* = 0.030), and all vision-only models (all *P* < 0.01; Fig. 4a, e). Under the same 5-fold cross-validation setting, Prost-LM also showed robust and consistent performance for csPCa diagnosis (Supplementary Fig. 1b,d), yielding a mean AUC of 0.956 (95% CI: 0.945–0.966) across all 5 folds. The narrow confidence intervals across folds further indicate that the model’s csPCa detection performance is highly stable under varying data distributions. This performance advantage of Prost-LM was consistently observed across all external cohorts. Prost-LM achieved AUCs of 0.899 (FHYL), 0.819 (FHZU), and 0.811 (AHJU), significantly outperforming all other models (all *P* < 0.05; Fig. 4b–d, f–h). This robust, multi-center validation underscores the model’s reliability in identifying the most aggressive forms of PCa.

**Fig. 5:**
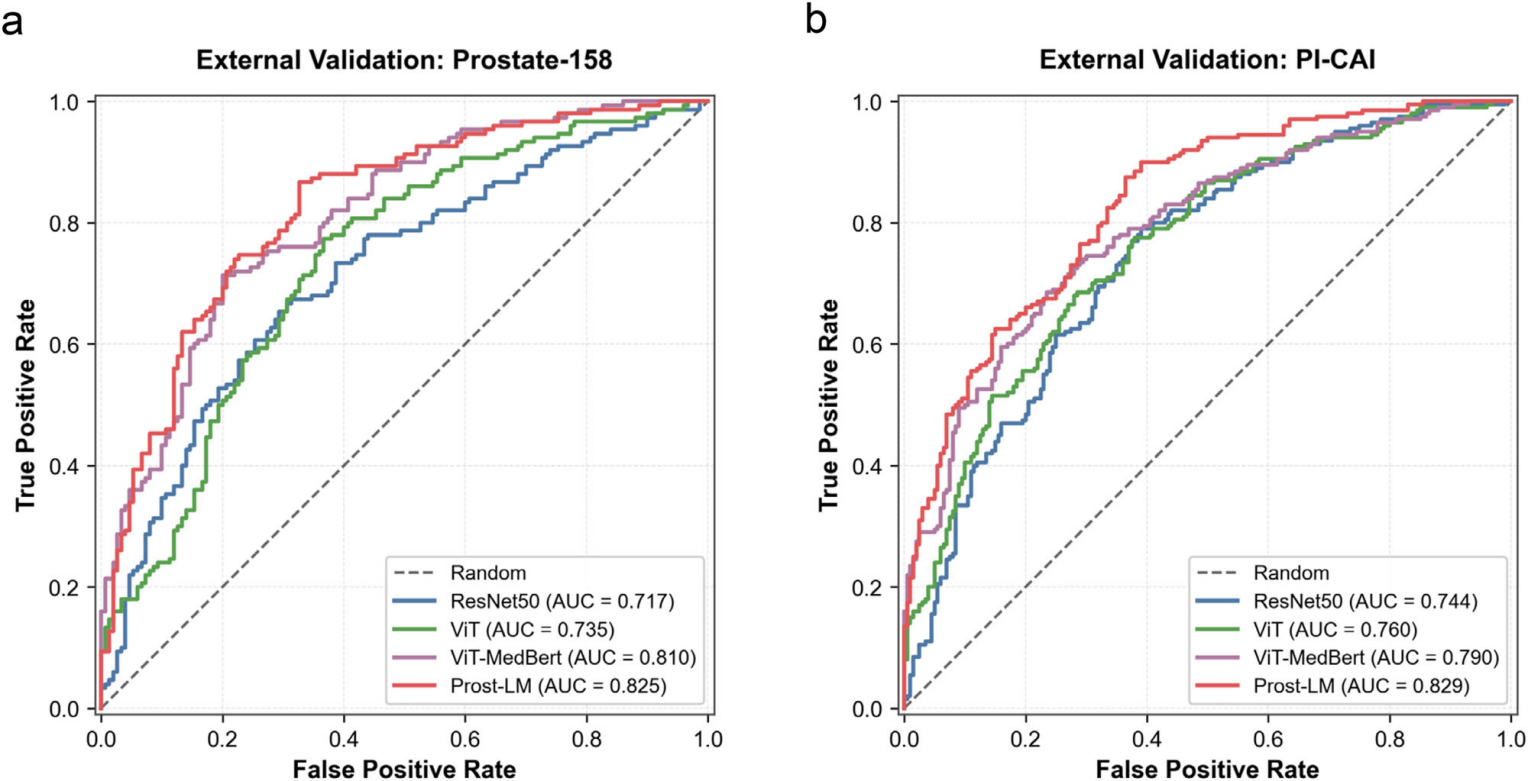
Performance benchmarking on external public datasets. **a**, **b** Receiver Operating Characteristic (ROC) curves evaluating the generalization performance of Prost-LM on two open-access external datasets: **a** Prostate-158 and (**b**) PI-CAI. Prost-LM (red line) consistently achieves the highest AUC compared to unimodal (ResNet50, ViT) and multimodal (ViT-MedBert) baselines, demonstrating robust generalization.

To further validate the generalizability of Prost-LM, we evaluated the model on two publicly open-access datasets: PI-CAI and Prostate-158. As shown in Fig. 5, Prost-LM achieved an AUC of 0.829 on PI-CAI and 0.825 on Prostate-158, consistently outperforming unimodal baselines (ResNet50, ViT) and the multimodal baseline ViT-MedBert (AUC = 0.790 and 0.810, respectively). These results confirm that Prost-LM learns robust, transferable diagnostic features rather than merely memorizing internal data distributions.

In the internal validation cohort (n=553), Prost-LM at a cut-off of 79.0% demonstrated remarkable potential. Furthermore, the safety of this approach was underscored by an excellent negative predictive value (NPV) of 94.7% (Table 1). To confirm the generalizability of Prost-LM, we applied the model to the three external cohorts (Table 1). The model consistently maintained high sensitivity (94.4–95.0%) and strong NPVs (85.5–94.7%) across all datasets.

**Table 1:** Performance of Prost-LM in the internal validation cohort, FHYL, FHZU, and AHJU cohorts at about 95% sensitivity

Cohort	Clinical diagnosis		Total	Performance, %
	csPCa	Benign+non-csPCa		
Internal cohort, cut-off value= 79.0%				
Prost-LM probability > cut point	257	34	291	Sensitivity=94.8
Prost-LM probability < = cut point	14	248	262	Specificity=87.9
Total	271	282	553	PPV=88.3, NPV=94.7
FHYL cohort, cut-off value= 13.1%				
Prost-LM probability > cut point	190	161	351	Sensitivity=95.0
Prost-LM probability < = cut point	10	118	128	Specificity=42.3
Total	200	279	479	PPV=54.1, NPV=92.2
FHZU Cohort, cut-off value= 24.8%				
Prost-LM probability > cut point	34	34	68	Sensitivity=94.4
Prost-LM probability < = cut point	2	23	25	Specificity=40.4
Total	36	57	93	PPV=50.0, NPV=92.0
AHJU Cohort, cut-off value= 8.3%				
Prost-LM probability > cut point	190	336	526	Sensitivity=94.5
Prost-LM probability < = cut point	11	65	76	Specificity=16.2
Total	201	401	602	PPV=36.1, NPV=85.5

*csPCa* Clinically significant prostate cancer, *non-csPCa* non-clinically significant prostate cancer, *NPV* Negative predictive value, *PPV* Positive predictive value.

**Fig. 6:**
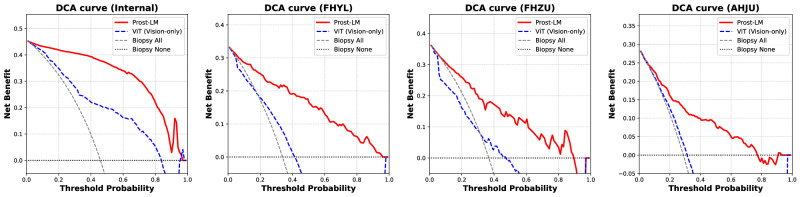
Decision curve analysis demonstrating the improved clinical utility of the multimodal Prost-LM. Decision curve analysis (DCA) for the Prost-LM models across four independent cohorts: internal validation, FHYL, FHZU, and AHJU. The y-axis represents the net benefit, which balances the benefit of detecting true positives against the harm of unnecessary interventions for false positives. The *x*-axis represents the threshold probability, which reflects a clinician’s preference for intervention. The solid red line (Prost-LM Multimodal) consistently shows a greater net benefit compared to the dashed blue line (Prost-LM Vision-only). Both models outperform the default strategies of biopsying all patients (gray dashed line) or none (black dotted line).

To further evaluate the clinical value of Prost-LM, we performed DCA to assess its net benefit across a continuous range of decision thresholds. As shown in Fig. 6, the DCA revealed that our multi-modal Prost-LM consistently provided a higher net benefit than both the vision-only model (diagnosis with only MRI) and the default strategies of biopsying all or no patients. This advantage was evident across a wide and clinically relevant range of threshold probabilities in all four cohorts.

### Ablation study and comparison with traditional machine learning

**Fig. 7:**
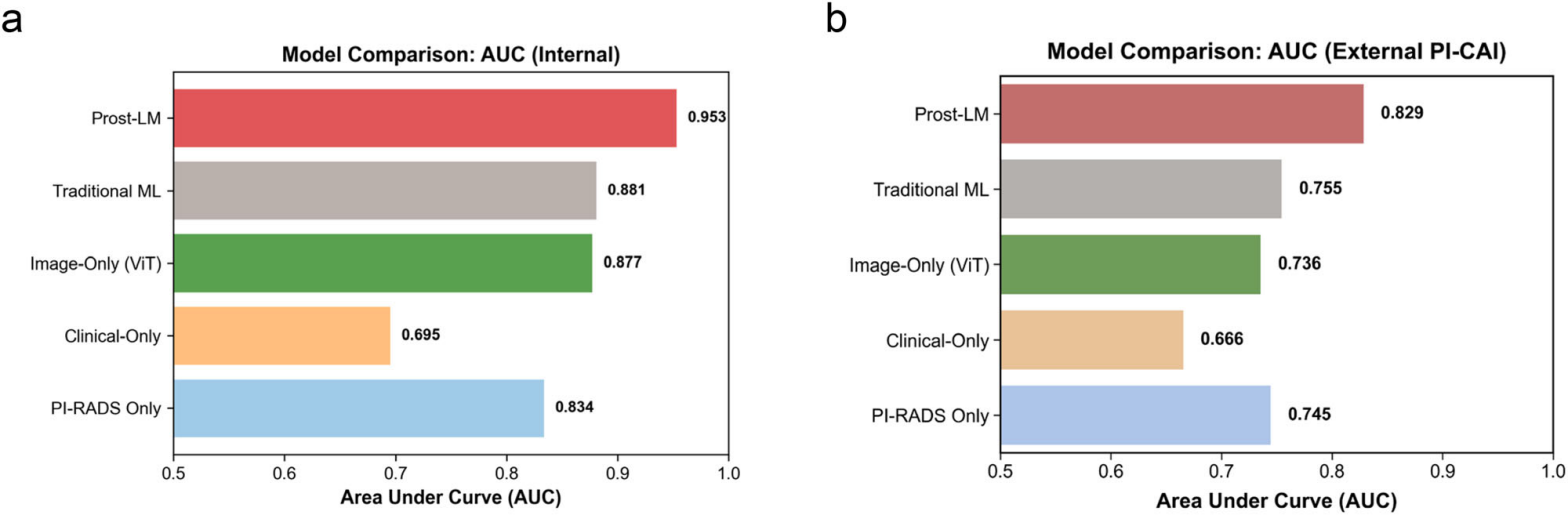
Comprehensive ablation study and performance comparison. **a**, **b** Comparison of Area Under the Curve (AUC) across different model architectures. Specifically, **a** displays the performance on the internal validation cohort, while (**b**) shows the performance on the external PI-CAI dataset. Prost-LM (Red) consistently outperforms other baselines. The multimodal approach of Prost-LM demonstrates improved robustness compared to single-modal baselines (Image-Only or Clinical-Only) and the traditional machine learning method.

To justify the architectural complexity of Prost-LM, we conducted comprehensive ablation studies comparing against single-modality baselines and traditional machine learning methods. We constructed the following baseline models: (1) “PI-RADS Only” logistic regression; (2) “Clinical-Only” model using structured clinical features excluding MRI; (3) “Image-Only” Vision Transformer; and (4) “Traditional ML” XGBoost ensemble trained on the same combined feature set as Prost-LM but without the LLM backbone. As presented in Fig. 7, Prost-LM significantly outperformed all single-modality baselines. The “PI-RADS Only” model achieved an AUC of 0.834 (internal) and 0.745 (PI-CAI), substantially lower than Prost-LM’s AUC of 0.953 (internal) and 0.829 (PI-CAI). Notably, Traditional ML achieved AUCs of 0.881 (internal) and 0.755 (PI-CAI), representing performance gaps of +7.2% and +7.4%, respectively, compared to Prost-LM. These results demonstrate that the LLM-based architecture delivers meaningful diagnostic gains by capturing deep, complex, cross-modal interactions that conventional methods cannot adequately model.

To demonstrate Prost-LM’s ability to model complex non-linear interactions, we analyzed confusion matrices comparing Traditional ML and Prost-LM (Supplementary Fig. 2). Prost-LM reduced False Negatives from 56 (28.0%) to 25 (12.5%) compared to Traditional ML, confirming that the LLM architecture correctly identifies high-risk patients that linear models overlook. False Positives were also better managed, validating that the model does not merely reproduce radiologist bias but captures genuine diagnostic signals.

### Comparison with radiologist performance

**Fig. 8:**
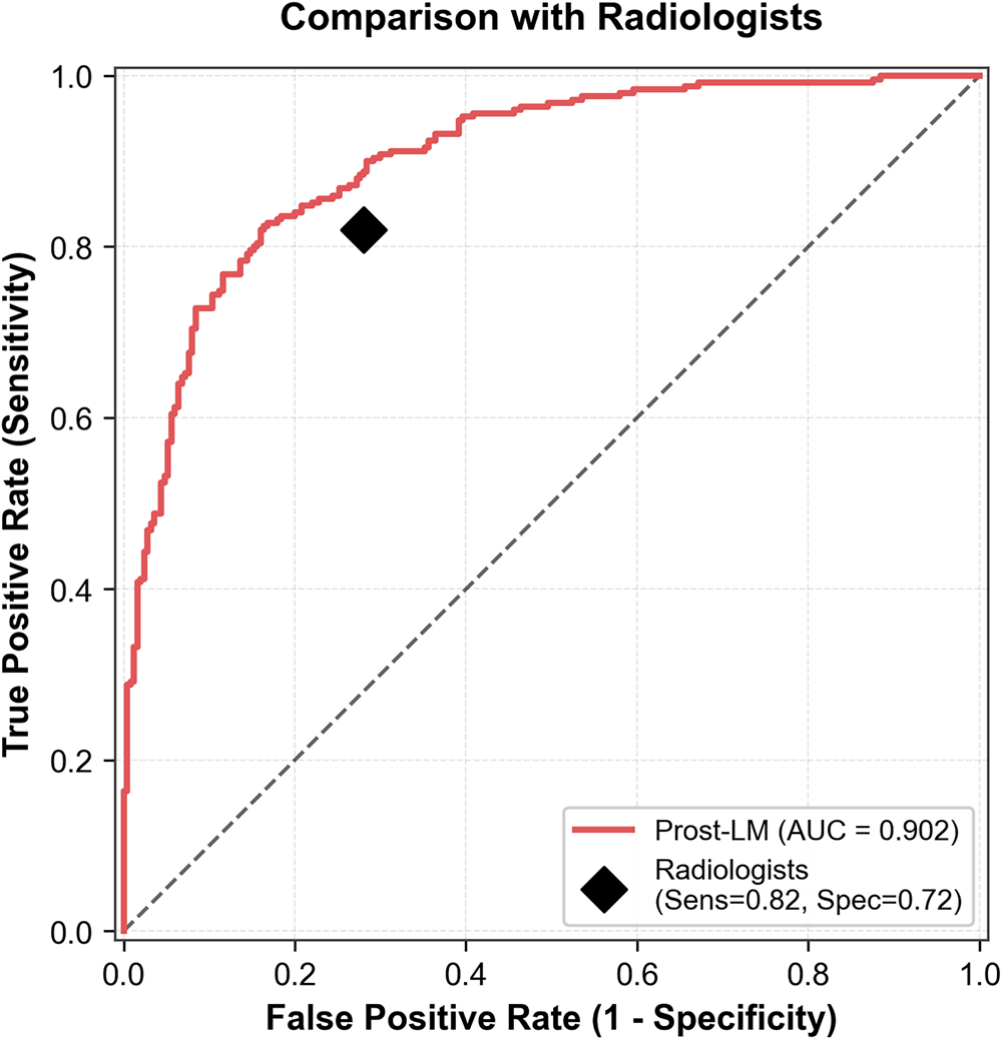
Performance benchmarking against expert radiologist consensus. The Receiver Operating Characteristic (ROC) curve compares the diagnostic performance of Prost-LM (red solid line) with a panel of human experts. The black diamond (◇) represents the operating point of three senior radiologists (each with > 10 years of experience) derived using a majority voting strategy.

To robustly benchmark Prost-LM against human expertise, we conducted a retrospective multi-reader study on 200 patients from the internal test set. The reading panel consisted of three senior board-certified radiologists, each possessing over 10 years of experience in urogenital imaging. The radiologists independently reviewed the MRI scans (T2WI, DWI, ADC) blinded to pathological outcomes. To derive a single robust human performance metric, we employed a majority voting strategy: the final diagnosis for each case was determined by the concordance of at least two out of the three readers. Under this rigorous setting, the expert panel achieved a sensitivity of 0.82 and specificity of 0.72. As illustrated in Fig. 8, the ROC curve of Prost-LM (AUC = 0.902) passes significantly above this expert consensus point (black diamond). This demonstrates that Prost-LM outperforms even a committee of senior experts, highlighting its value in reducing inter-reader variability and improving diagnostic accuracy.

### Qualitative attention analysis

**Fig. 9:**
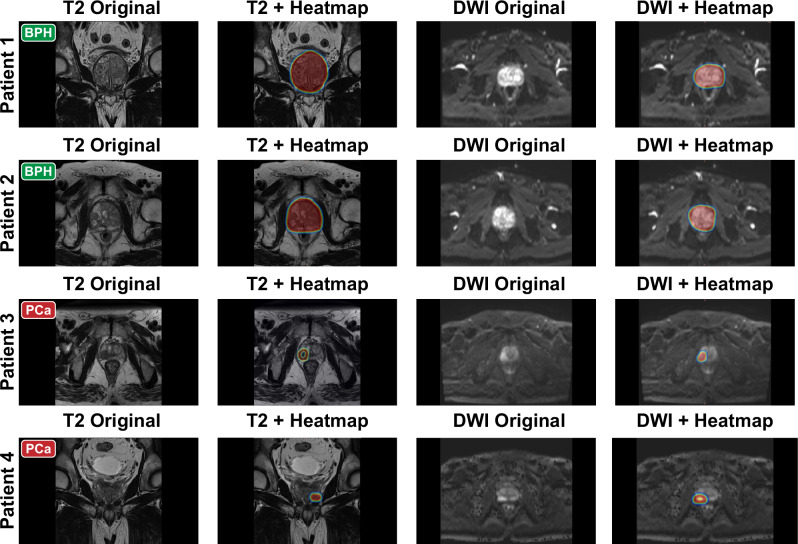
Qualitative analysis of model attention on physician-validated ROIs. Four cases are presented: Patients 1 & 2 (BPH) show diffuse attention on the transition zone, consistent with benign changes; Patients 3 & 4 (PCa) show focal, high-intensity attention on specific lesions in the peripheral zone. The heatmaps overlaying T2 and DWI images confirm the model’s ability to identify “patchy” abnormal regions.

We generated attention heatmaps for 4 representative cases (2 BPH, 2 PCa) validated by experienced radiologists (Fig. 9). In BPH cases, attention diffusely mapped to the transition zone, consistent with benign changes. In PCa cases, focal high-intensity attention localized to specific peripheral zone lesions, precisely corresponding with T2-hypointense and DWI-hyperintense regions identified by radiologists. These visualizations confirm Prost-LM’s ability to identify clinically relevant anatomical regions.

### Multimodal input representations

**Fig. 10:**
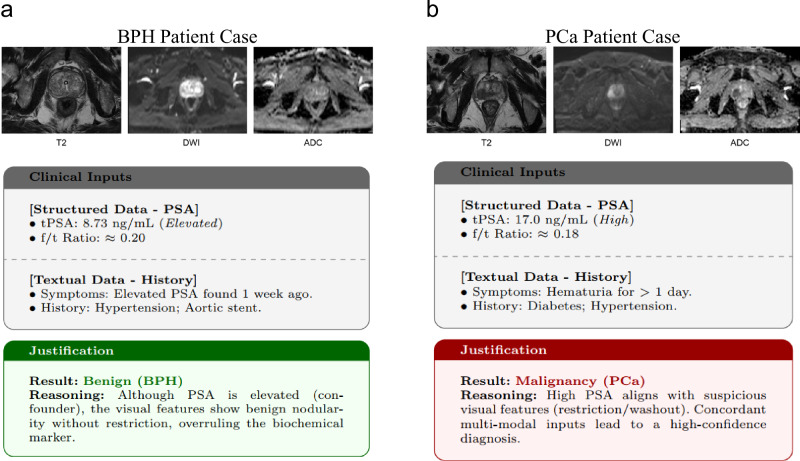
Visualization of multi-modal input representations. The figure displays the three specific input forms fed into the model: Visual (MRI), Structured (PSA), and Textual (History). **a** A BPH case where the model correctly identifies the condition as benign despite elevated PSA, by prioritizing visual evidence. **b** A PCa case where concordant high PSA and suspicious imaging confirm malignancy. This comparison demonstrates the necessity of integrating multiple data representations to resolve diagnostic conflicts.

To illustrate how Prost-LM integrates heterogeneous data, we visualized the three input modalities for representative BPH and PCa cases (Fig. 10). In the BPH case, despite elevated PSA (8.73 ng/mL), the model correctly identified benign disease by prioritizing visual evidence (nodularity without restriction). In the PCa case, concordant high PSA (17.0 ng/mL) and suspicious imaging confirmed malignancy. This comparison demonstrates that fusion of visual and textual representations is critical for resolving diagnostic conflicts when structured data alone is equivocal. Detailed logs of model interactions, including query-response pairs for representative cases, are provided in the supplementary material.

## Discussion

This study presents Prost-LM, a multimodal large language model framework that integrates heterogeneous clinical data, including MRI, PSA levels, and medical records, to advance PCa diagnosis. By leveraging the advanced feature modeling capability of large models, Prost-LM achieves significant improvements compared to previous MRI-based approaches and multi-modal small model. To our knowledge, Prost-LM is the first large multimodal model specific for this task. Its effectiveness stems from a unique knowledge-infusion strategy: it first learns foundational concepts from curated textbook image-text pairs and then masters complex diagnostic reasoning by training on real-world clinical image-report data, enabling a deep, synergistic understanding of PCa.

Our results reveal that Prost-LM achieves an AUC of 0.800–0.954 for PCa detection across seven tertiary hospitals. The model’s capability is further evidenced by the performance in distinguishing csPCa, where it attains an AUC of 0.811–0.955. Prost-LM represents a 13% (PCa) and 8% (csPCa) improvement over the previous approach, averaged over all cohorts. Prost-LM streamlines the diagnostic workflow by automating multimodal data integration. It reduces the expert assessment time from an average of 4.2 min to under 2 s per case, an acceleration of over 125-fold. This capability for high-throughput, expert-level analysis directly addresses a major bottleneck in timely patient care.

Prost-LM achieves significantly higher diagnostic accuracy than existing deep learning models, improving PCa AUC by 7.8% over MRI-only model ViT, and 3.4% compared to small image-text joint model ViT-MedBert, averaged on all cohorts. The MRI-only baseline ViT relies exclusively on MRI-derived biomarkers such as T2 signal intensity ratios and tumor boundary irregularity in the peripheral zone. However, in clinically challenging cases with equivocal MRI findings (e.g., small lesions < 5 mm, periprostatic inflammation mimicking tumor margins), these visual cues exhibit low discriminative power. In contrast, Prost-LM effectively integrates complementary evidence from PSA metric and complaint to resolve diagnostic ambiguity.

As for image-text joint models, ViT-MedBert adopts coarse-grained fusion strategies that concatenate global image features with pooled text embeddings. This approach overlooks fine-grained cross-modal interactions essential for medical diagnosis. For example, consider a case where an MRI reveals a PI-RADS 3 lesion, which is equivocal. A conventional model might struggle. However, if the clinical notes describe a patient with a “rapidly rising PSA trend over 6 months”, Prost-LM could comprehensively reason the ambiguous lesion visual features with the high-risk medical context.

While traditional ML models remain more computationally efficient, their performance plateaus due to reliance on linear or shallow non-linear relationships. Our ablation study demonstrates that Prost-LM outperforms XGBoost by +7.2% (internal) and +7.4% (PI-CAI), providing strong empirical evidence that the LLM architecture captures deep cross-modal interactions. This substantial performance improvement justifies the adoption of Prost-LM despite higher computational demands.

We acknowledge the performance gap between internal validation (AUC = 0.954) and external cohorts (AUC = 0.800–0.899). This discrepancy primarily reflects domain shift–a pervasive challenge in medical AI–rather than model overfitting. Our validation on public benchmarks (PI-CAI: AUC = 0.829; Prostate-158: AUC = 0.825) confirms that Prost-LM learns robust, transferable features. The performance drop stems from clinical heterogeneity: external cohorts utilize diverse MRI scanners (different vendors, 1.5T vs. 3.0T) and acquisition parameters. Importantly, Prost-LM maintains stronger performance than other approaches across all settings, demonstrating that multimodal clinical knowledge effectively mitigates visual domain shift compared to vision-only models.

The low specificity observed in the AHJU cohort (16.2%) reflects our strategic prioritization of sensitivity over specificity in the presence of significant domain shift. To maintain ~ 95% sensitivity for cancer detection, we lowered the classification threshold (8.3% vs. 79.0% internally), resulting in more BPH cases flagged as positive. Crucially, Prost-LM is designed as a Second Reader” or triage tool, not a standalone decision-maker. The radiologist acts as final gatekeeper, incorporating qualitative judgment to rule out obvious false positives before biopsy recommendations. This "Human-in-the-loop” verification is essential to prevent overdiagnosis in external cohorts with significant domain shifts.

To provide insight into the model’s internal reasoning, we analyzed its step-wise diagnostic process on representative clinical cases (Fig. 10). The model first demonstrates a strong command of anatomical localization by correctly identifying suspicious lesions on T2-weighted images. It then progresses to functional characterization, accurately associating these lesions with high-risk features like restricted diffusion on ADC maps. This sequential, multi-modal reasoning is highly significant because it mirrors the diagnostic paradigm of expert radiologists. Also, we have compiled a detailed log of 10 distinct patient instances in the Supplementary Note 1. It indicates that Prost-LM has learned not just to correlate inputs with outputs, but to follow a clinically logical pathway of inquiry. This behavior provides qualitative yet powerful evidence that the model’s improved performance is built upon a learned foundation of clinical knowledge. This ability to derive clinical reasoning directly from data is crucial for establishing its trustworthiness in real-world applications.

Also, the model addresses a pervasive challenge in urological practice: the high rate of false positives inherent in standard PI-RADS assessments. As evidenced by the confusion matrix analysis, Prost-LM significantly improves specificity compared to traditional machine learning methods. This reduction in false positives is clinically useful; it implies that deploying such a model could substantially reduce the number of unnecessary biopsies for benign conditions, such as Benign Prostatic Hyperplasia (BPH), which often confounds MRI interpretation. The fact that the model’s operating point encompasses that of senior radiologists further suggests its potential as a reliable second reader, capable of mitigating inter-reader variability and standardizing diagnostic accuracy across varying levels of clinical expertise.

Furthermore, the model’s decision-making process exhibits a high degree of biological plausibility, essential for clinical adoption. The distinct attention patterns observed in our qualitative analysis provide assurance that the improved performance is not driven by confounding background artifacts. The model autonomously learned to distinguish the diffuse signal enhancement typical of transition zone BPH from the focal, high-intensity signatures of clinically significant carcinoma. This alignment between the model’s “visual focus” and established pathological characteristics suggests that Prost-LM has successfully encoded meaningful radiological semantics.

## Methods

### Overview of the proposed framework

This study proposes a comprehensive two-step framework based on multi-modal LLMs (MLLMs), aimed at advancing PCa diagnosis through the integration of multi-modal clinical data. The framework incorporates both 10,000 image-text pairs crafted from the textbook and 5000 doctor-annotated Chain-of-Thought (CoT) examples that formalize diagnostic reasoning patterns in PCa assessment. The first step involves a global-local visual-language pretraining stage, where we tailor the Contrastive Language-Image Pretraining (CLIP) model to embed medical knowledge effectively into the visual encoder. The second step integrates this pretrained visual model with the Qwen-7B-VL large language model via a cross-attention bridge.

### Data preprocessing

#### MR images

The dataset comprised MRI scans from 3940 patients across seven hospitals. These images were procured utilizing apparatuses furnished by GE Healthcare and Siemens Healthineers. The imaging protocol included: T2WI, T2FS, DWI, and ADC mapping. Note that while the raw clinical database contained comprehensive imaging data including DCEI for many patients, the specific input modalities selected for model development were restricted to T2WI, T2FS, DWI, and ADC sequences to align with the biparametric MRI (bpMRI) protocol. Each image was resized to a uniform resolution of 256 × 256 pixels to standardize input dimensions for the model. To account for differences in intensity scales across scanners, we applied z-score normalization, calculated as *z* = (*x* − *μ*)/*σ*, where *x* is the pixel intensity, *μ* is the mean intensity, and *σ* is the standard deviation across the dataset.

#### Clinical reports

Accompanying the MR images, we collected detailed radiology reports that described lesion characteristics (e.g., size, shape, margins), prostate zones (e.g., peripheral, transitional), and Prostate Imaging-Reporting and Data System (PI-RADS) scores. These reports underwent extensive preprocessing: text was tokenized using a natural language processing (NLP) toolkit, stop words (e.g., “the”, “and”) were removed to reduce noise, and clinical terms were normalized using a medical ontology (e.g., UMLS). This preprocessing ensured that the textual data was structured and focused on diagnostically relevant information.

#### CoT annotations

Additionally, we collect 3000 doctor-annotated Chain-of-Thought (CoT) examples that explicitly document diagnostic reasoning pathways (e.g., “Restricted diffusion in peripheral zone → PI-RADS 4 lesion → recommend targeted biopsy”). CoT annotations were structured into *observation → interpretation → recommendation* triples for supervised fine-tuning.

### Global-local visual-language pretraining

To bridge the gap between general-purpose vision-language models and the specialized domain of medical diagnostics, we extensively modified the vanilla CLIP framework by introducing several novel adaptations. These improvements include (1) extracting the local medical entities from the original MRI report with background noises (Medical Entity-Aware Contrastive Learning), and (2) finding the MRI regions most related to the MRI report description, and emphasizing the visual-language learning of these regions (Local Anatomy-Aware Contrastive Learning).

Our first innovation augments the CLIP model by incorporating medical entity embeddings. We extracted key medical entities (e.g., “lesion”, “PI-RADS score”, “prostate zone”) from clinical reports using a pretrained BioBERT model, fine-tuned on a corpus of radiology texts. These entities were embedded into a high-dimensional space and integrated into the contrastive learning process. Unlike the standard CLIP, which aligns images and text globally, our approach emphasizes clinically significant terms, enhancing the model’s focus on diagnostic relevance.

The standard CLIP loss is:

1 $${{\mathcal{L}}}_{{\rm{C}}{\rm{L}}{\rm{I}}{\rm{P}}}=-\frac{1}{N}\mathop{\sum }\limits_{i=1}^{N}\log \frac{\exp ({\rm{s}}{\rm{i}}{\rm{m}}({v}_{i},{t}_{i})/\tau )}{{\sum }_{j=1}^{N}\exp ({\rm{s}}{\rm{i}}{\rm{m}}({v}_{i},{t}_{j})/\tau )},$$

where *v*_*i*_ is the image embedding, *t*_*i*_ is the text embedding, $sim(\cdot )$ denotes cosine similarity, *τ* is a learnable temperature parameter, and *N* is the batch size.

We redefine the similarity function to include medical entity embeddings *e*_*i*_:

2 $${{\mathrm{sim}}}^{{\prime} }({v}_{i},{t}_{i})={\mathrm{sim}}({v}_{i},{t}_{i})+{\lambda }_{1}\cdot {\mathrm{sim}}({v}_{i},{e}_{i}),$$

where *λ*_1_ = 0.3 balances the contribution of entity embeddings. This modification ensures that the model prioritizes alignments between image features and critical medical concepts.

To further enhance the CLIP model for medical imaging, we introduced a sophisticated region-specific cross-modal attention mechanism, inspired by the Multi-Granularity Cross-modal Alignment (MGCA) approach^31^. Prostate MR images often contain critical localized regions (e.g., lesions in the peripheral zone) that are pivotal for diagnosis. To capture these, we employed a dynamic attention strategy that leverages both image and text embeddings to focus on diagnostically relevant areas.

Instead of relying on a fixed grid, we implemented a cross-modal attention mechanism where the clinical report’s text embedding guides the selection of important image patches. Each 256 × 256 MR image was divided into a 16 × 16 grid, resulting in 256 patches. For each patch, we computed an attention weight based on its relevance to the text description, ensuring the model prioritizes regions mentioned in the report.

Let *v*_*i*,*p*_ denote the embedding of the *p*-th image patch for the *i*-th sample, and *t*_*i*_ represent the text embedding of the corresponding clinical report. We calculated the attention weight *a*_*p*_ for each patch as:

3 $${a}_{p}=\frac{\exp ({\rm{s}}{\rm{i}}{\rm{m}}({v}_{i,p},{t}_{i}))}{{\sum }_{q=1}^{P}\exp ({\rm{s}}{\rm{i}}{\rm{m}}({v}_{i,q},{t}_{i}))},$$

where $sim(\cdot )$ is the cosine similarity, and *P* = 256 is the number of patches. This softmax-normalized weight reflects how well each patch aligns with the report’s description.

To capture both local and global contexts, we incorporated multi-scale features by applying the attention mechanism at different resolutions. Specifically, we generated patch embeddings at two scales: the original 16 × 16 grid and a coarser 8 × 8 grid. For the coarser grid, each patch covers a larger area, providing contextual information. The final image embedding ${v}_{i}^{{\prime} }$ is a weighted sum of patch embeddings from both scales:

4 $${v}_{i}^{{\prime} }=\mathop{\sum }\limits_{s=1}^{S}\mathop{\sum }\limits_{p=1}^{{P}_{s}}{a}_{s,p}\cdot {v}_{i,s,p},$$

where *S* = 2 scales, *P*_*s*_ is the number of patches at scale *s*, and *a*_*s*,*p*_ is the attention weight for patch *p* at scale *s*.

The region-specific cross-modal attention mechanism is seamlessly integrated into the Contrastive Language-Image Pretraining (CLIP) framework by replacing the standard image embedding with the weighted, multi-scale image embedding ${v}_{i}^{{\prime} }$. This ensures that the model aligns the most diagnostically relevant regions of the prostate MR images with the corresponding clinical report text, enhancing its ability to capture critical pathological features.

The standard CLIP contrastive loss is defined as:

5 $${{\mathcal{L}}}_{{\rm{C}}{\rm{L}}{\rm{I}}{\rm{P}}}=-\frac{1}{N}\underset{i=1}{\overset{N}{\sum \left[\log \frac{\exp (\mathrm{sim}({v}_{i},{t}_{i})/\tau )}{{\sum }_{j=1}^{N}\exp (\mathrm{sim}({v}_{i},{t}_{j})/\tau )}+\log \frac{\exp (\mathrm{sim}({t}_{i},{v}_{i})/\tau )}{{\sum }_{j=1}^{N}\exp (\mathrm{sim}({t}_{i},{v}_{j})/\tau )}\right]}},$$

where *v*_*i*_ is the image embedding, *t*_*i*_ is the text embedding, $sim(\cdot )$ denotes cosine similarity, *τ* is a learnable temperature parameter, and *N* is the batch size.

In our enhanced framework, we replace *v*_*i*_ with the region-specific multi-scale embedding ${v}_{i}^{{\prime} }$, which is computed as:

6 $${v}_{i}^{{\prime} }=\mathop{\sum }\limits_{s=1}^{S}\mathop{\sum }\limits_{p=1}^{{P}_{s}}{a}_{s,p}\cdot {v}_{i,s,p},$$

where *S* = 2 represents the two scales (16 × 16 and 8 × 8 grids), *P*_*s*_ is the number of patches at scale *s*, *v*_*i*,*s*,*p*_ is the embedding of patch *p* at scale *s*, and *a*_*s*,*p*_ is the attention weight derived from the cross-modal similarity:

7 $${a}_{s,p}=\frac{\exp ({\mathrm{sim}}({v}_{i,s,p},{t}_{i}))}{{\sum }_{q=1}^{{P}_{s}}\exp ({\mathrm{sim}}({v}_{i,s,q},{t}_{i}))}.$$

To further incorporate medical priors, we integrate medical entity embeddings *e*_*i*_ (as described in the Medical Entity-Aware Contrastive Learning section). The similarity function is modified to:

8 $${{\mathrm{sim}}}^{{\prime} }({v}_{i}^{{\prime} },{t}_{i})={\bf{\mathrm{sim}}}({v}_{i}^{{\prime} },{t}_{i})+{\lambda }_{1}\cdot {\mathrm{sim}}({v}_{i}^{{\prime} },{e}_{i}),$$

where *λ*_1_ = 0.3 balances the contribution of entity embeddings.

The enhanced CLIP loss, incorporating both the region-specific embedding and medical entity alignment, is:

9 $${{\mathcal{L}}}_{{\rm{e}}{\rm{n}}{\rm{h}}{\rm{a}}{\rm{n}}{\rm{c}}{\rm{e}}{\rm{d}}}=-\frac{1}{N}\mathop{\sum }\limits_{i=1}^{N}\left[\log \frac{\exp ({\mathrm{sim}}^{{\prime} }({v}_{i}^{{\prime} },{t}_{i})/\tau )}{{\sum }_{j=1}^{N}\exp ({\mathrm{sim}}^{{\prime} }({v}_{i}^{{\prime} },{t}_{j})/\tau )}+\log \frac{\exp ({\mathrm{sim}}^{{\prime} }({t}_{i},{v}_{i}^{{\prime} })/\tau )}{{\sum }_{j=1}^{N}\exp ({\mathrm{sim}}^{{\prime} }({t}_{i},{v}_{j}^{{\prime} })/\tau )}\right],$$

This loss ensures that the model learns to align the text-guided, multi-scale image embeddings with clinical reports while emphasizing medically relevant features through entity embeddings. The attention mechanism is fully differentiable, allowing end-to-end training that adaptively focuses on critical regions based on both visual and textual cues.

As for Medical Entity Encoder, A separate BioBERT instance generates embeddings for extracted medical entities. This encoder mirrors the text encoder’s architecture but is fine-tuned specifically for entity recognition and embedding. The resulting entity embeddings are fixed during pretraining to stabilize the contrastive learning process.

As for image encoder, we utilized a Vision Transformer (ViT-B/16) pretrained on ImageNet, adapted for MR images by modifying the input layer to accept two channels (T2WI and DWI). The ViT comprises 12 transformer layers, each with 12 attention heads and a hidden dimension of 768. Images are divided into 16 × 16 patches, yielding 256 patch embeddings per image, plus a [CLS] token. During pretraining, the encoder incorporates region-specific attention weights, enhancing its focus on diagnostically relevant areas. The image-level features within the same slice are passed through a temporal attention block to obtain the slice-level feature.

As for text encoder, we adopted a BioBERT model, which is pretrained on PubMed abstracts and fine-tuned on radiology reports, serves as the text encoder. It consists of 12 transformer layers, 12 attention heads, and a hidden dimension of 768. The encoder processes tokenized clinical reports, producing a contextualized embedding for the [CLS] token, which represents the entire report.

As for training details, we utilized the AdamW optimizer^32^ for model training, which is well-suited for large-scale models due to its adaptive learning rates and effective weight decay regularization. The initial learning rate was set to 1 × 10^−4^ for the vision encoder and 5 × 10^−5^ for the text encoder, reflecting the common practice of using a slightly lower learning rate for pre-trained language model components. We employed a cosine annealing learning rate scheduler with a warm-up period of 5 epochs. The weight decay was set to 1 × 10^−2^. The model was trained for a total of 100 epochs with a batch size of 128 (32 per GPU). The model checkpoint with the lowest validation loss was selected for all downstream evaluation tasks.

### Integration with Qwen-7B-VL Large Language Model

The pretrained visual encoder is integrated with Qwen-7B-VL through a dual-stage adaptation protocol designed to align medical reasoning:

Stage 1: Medical Knowledge Injection via CoT-LoRA. We inject domain-specific knowledge into Qwen’s language module using Low-Rank Adaptation (LoRA) on the **5,000 CoT annotations**. LoRA matrices are applied to attention weights:

10 $${W}^{{\prime} }=W+\alpha \,B{A}^{\top },$$

where *W* are original weights, $A\in {{\mathbb{R}}}^{d\times r}$, $B\in {{\mathbb{R}}}^{r\times k}$ are low-rank adapters (*r* = 8), and *α* = 0.5 scales updates. Training uses next-token prediction loss on CoT sequences, enabling the model to generate clinically plausible reasoning chains.

The LoRA adapters were configured with a rank of *r* = 8 and applied to the query and value matrices of the self-attention blocks. The scaling factor *α* was set to 16. For this stage, we used the AdamW optimizer with an initial learning rate of 2 × 10^−5^, a weight decay of 0.01, and a batch size of 16 (4 per GPU). The model was fine-tuned for 3 epochs, optimizing a standard next-token prediction (cross-entropy) loss on the CoT sequences. During this stage, the visual encoder of Qwen-7B-VL remained frozen.

Stage 2: Cross-Modal Alignment via Attention Bridge. A trainable cross-attention bridge connects our pretrained visual encoder (*f*_*θ*_) with Qwen’s visual encoder (*g*_*ϕ*_):

11 $$\begin{array}{rcl}Q & = & {\rm{L}}{\rm{i}}{\rm{n}}{\rm{e}}{\rm{a}}{\rm{r}}({g}_{\phi }({x}_{{\rm{i}}{\rm{m}}{\rm{g}}})),\\ K,V & = & {\rm{L}}{\rm{i}}{\rm{n}}{\rm{e}}{\rm{a}}{\rm{r}}({f}_{\theta }({x}_{{\rm{i}}{\rm{m}}{\rm{g}}})),\\ {\rm{B}}{\rm{r}}{\rm{i}}{\rm{d}}{\rm{g}}{\rm{e}}({x}_{{\rm{i}}{\rm{m}}{\rm{g}}}) & = & {\rm{s}}{\rm{o}}{\rm{f}}{\rm{t}}{\rm{m}}{\rm{a}}{\rm{x}}\left(\frac{Q{K}^{\top }}{\sqrt{{d}_{k}}}\right)V.\end{array}$$

The bridge is optimized using instruction tuning on multimodal medical QA pairs. This enables seamless fusion between domain-specific visual features and Qwen’s semantic space.

The bridge was trained for 10 epochs using the AdamW optimizer, a higher learning rate of 1 × 10^−4^ (as it was trained from scratch), and a batch size of 32. The objective was to minimize the cross-entropy loss on the answer tokens, conditioned on the fused visual features and the question prompt. A cosine learning rate scheduler with a linear warm-up over the first epoch was employed.

### Ethics approval and data acquisition

The study protocol was approved by the ethics committees of all participating centers: Shanghai Jiao Tong University Ninth People’s Hospital (Ethics ID: SH9H-2025-T157-1), Jiaxing First Hospital (Ethics ID: 2025-LP-447), The First Affiliated Hospital of Soochow University (Ethics ID: 2025532), The First Affiliated Hospital of Zhengzhou University (Ethics ID: 2025-KY-0740-002), The First People’s Hospital of Yulin City (Ethics ID: YLSY-IRB-SR-2025150), The First Affiliated Hospital of Naval Medical University (Ethics ID: CHEC2026-014), and The First People’s Hospital of Yancheng City (Ethics ID: 2024-K(YJ)-032). Informed consent was obtained from all patients for the use of their medical data in research. All patient data were anonymized prior to analysis in accordance with the Declaration of Helsinki. This multicenter retrospective cohort study included consecutive patients evaluated at each site between January 2018 and December 2024 who met the following criteria. Inclusion Criteria: (1) Clinically diagnosed prostate cancer (PCa), benign prostatic hyperplasia (BPH), or other benign prostatic conditions; (2) MRI sequences, including T2-weighted imaging (T2WI), fat-suppressed T2WI (T2FS), diffusion-weighted imaging (DWI), and apparent diffusion coefficient (ADC) mapping; (3) Comprehensive clinical data, including serum prostate-specific antigen (PSA) level, detailed medical history (e.g., prior prostate interventions, medication use), and chief complaint. Exclusion Criteria: (1) Suboptimal MRI quality precluding reliable quantitative analysis (e.g., motion artifacts, severe metal artifacts); (2) History of prostate biopsy, prior prostate surgery, radiotherapy, or endocrine therapy before the MRI acquisition; (3) Severe systemic diseases affecting imaging or prognosis assessment (e.g., heart failure, end-stage renal disease).

## Supplementary information

Supplementary Information

## Data Availability

The datasets generated and/or analyzed during the current study are not publicly available due to patient privacy regulations and institutional ethical restrictions but are available from the corresponding author on reasonable request and ethics committee approval.

## Code availability

The custom Python scripts used for MRI feature extraction and model training are available from the corresponding author upon reasonable request. Analyses were performed using Python 3.10, PyTorch 2.5.1, transformers 4.49.0, qwen-vl-utils 0.0.8, NumPy 1.24.3, pandas 2.0.3, SciPy 1.10.1, matplotlib 3.7.2, seaborn 0.12.2, and OpenCV-Python 4.8.1.78. No proprietary or restricted software was used.

## References

[1] Bray, F. et al. Global cancer statistics 2022: Globocan estimates of incidence and mortality worldwide for 36 cancers in 185 countries. *Cancer J. Clin.* **74**, 229–263 (2024). 10.3322/caac.21834 38572751

[2] Siegel, R. L., Miller, K. D., Fuchs, H. E. & Jemal, A. Cancer statistics, 2022. *Cancer J. Clin.* **72**, 7–33 (2022). 10.3322/caac.21708 35020204

[3] Budäus, L. et al. Functional outcomes and complications following radiation therapy for prostate cancer: a critical analysis of the literature. *Eur. Urol.* **61**, 112–127 (2012). 22001105 10.1016/j.eururo.2011.09.027

[4] Weinreb, J. C. et al. Pi-rads prostate imaging–reporting and data system: 2015, version 2. *Eur. Urol.* **69**, 16–40 (2016). 26427566 10.1016/j.eururo.2015.08.052 PMC6467207

[5] Woo, S., Suh, C. H., Kim, S. Y., Cho, J. Y. & Kim, S. H. Diagnostic performance of prostate imaging reporting and data system version 2 for detection of prostate cancer: a systematic review and diagnostic meta-analysis. *Eur. Urol.* **72**, 177–188 (2017). 28196723 10.1016/j.eururo.2017.01.042

[6] Westphalen, A. C. et al. Variability of the positive predictive value of pi-rads for prostate MRI across 26 centers: experience of the Society of Abdominal Radiology prostate cancer disease-focused panel. *Radiology* **296**, 76–84 (2020). 32315265 10.1148/radiol.2020190646 PMC7373346

[7] Emmett, L. et al. The additive diagnostic value of prostate-specific membrane antigen positron emission tomography computed tomography to multiparametric magnetic resonance imaging triage in the diagnosis of prostate cancer (primary): a prospective multicentre study. *Eur. Urol.* **80**, 682–689 (2021). 34465492 10.1016/j.eururo.2021.08.002

[8] Wang, N. N. et al. Applying the precision approach in biopsy naive and previously negative prostate biopsy patients. In *Urologic Oncology: Seminars and Original Investigations*, vol. 37, 530–e19 (Elsevier, 2019). 10.1016/j.urolonc.2019.05.002 31151788

[9] Stabile, A. et al. Multiparametric MRI for prostate cancer diagnosis: current status and future directions. *Nat. Rev. Urol.* **17**, 41–61 (2020). 31316185 10.1038/s41585-019-0212-4

[10] Lilja, H. et al. Long-term prediction of prostate cancer up to 25 years before diagnosis of prostate cancer using prostate kallikreins measured at age 44 to 50 years. *J. Clin. Oncol.* **25**, 431–436 (2007). 17264339 10.1200/JCO.2006.06.9351

[11] Chen, S. et al. Machine learning-based models enhance the prediction of prostate cancer. *Front. Oncol.* **12**, 941349 (2022). 35875103 10.3389/fonc.2022.941349 PMC9299367

[12] Zhang, Y.-D. et al. An imaging-based approach predicts clinical outcomes in prostate cancer through a novel support vector machine classification. *Oncotarget* **7**, 78140 (2016). 27542201 10.18632/oncotarget.11293 PMC5363650

[13] Xiao, L.-H. et al. Prostate cancer prediction using the random forest algorithm that takes into account transrectal ultrasound findings, age, and serum levels of prostate-specific antigen. *Asian J. Androl.* **19**, 586–590 (2017). 27586028 10.4103/1008-682X.186884 PMC5566854

[14] Stenman, U.-H., Leinonen, J., Zhang, W.-M. & Finne, P. Prostate-specific antigen. In *Seminars in Cancer Biology*, vol. 9, 83–93 (Elsevier, 1999). 10.1006/scbi.1998.0086 10202130

[15] Litjens, G., Debats, O., Barentsz, J., Karssemeijer, N. & Huisman, H. Computer-aided detection of prostate cancer in MRI. *IEEE Trans. Med. imaging* **33**, 1083–1092 (2014). 24770913 10.1109/TMI.2014.2303821

[16] Ankerst, D. P. et al. A contemporary prostate biopsy risk calculator based on multiple heterogeneous cohorts. *Eur. Urol.* **74**, 197–203 (2018). 29778349 10.1016/j.eururo.2018.05.003 PMC6082177

[17] Saha, A. et al. Artificial intelligence and radiologists in prostate cancer detection on MRI (pi-cai): an international, paired, non-inferiority, confirmatory study. *Lancet Oncol.* **25**, 879–887 (2024). 38876123 10.1016/S1470-2045(24)00220-1 PMC11587881

[18] Liu, B. et al. Prediction of prostate cancer aggressiveness with a combination of radiomics and machine learning-based analysis of dynamic contrast-enhanced MRI. *Clin. Radiol.* **74**, 896–e1 (2019). 10.1016/j.crad.2019.07.011 31495546

[19] Abbasi, A. A. et al. Detecting prostate cancer using deep learning convolution neural network with transfer learning approach. *Cogn. Neurodyn.* **14**, 523–533 (2020). 32655715 10.1007/s11571-020-09587-5 PMC7334337

[20] Hamm, C. A. et al. Interactive explainable deep learning model informs prostate cancer diagnosis at MRI. *Radiology* **307**, e222276 (2023). 37039688 10.1148/radiol.222276

[21] Hiremath, A. et al. An integrated nomogram combining deep learning, prostate imaging–reporting and data system (PI-RADS) scoring, and clinical variables for identification of clinically significant prostate cancer on biparametric MRI: a retrospective multicentre study. *Lancet Digit. Health* **3**, e445–e454 (2021). 34167765 10.1016/S2589-7500(21)00082-0 PMC8261599

[22] Bacchetti, E. et al. A deep learning model integrating clinical and MRI features improves risk stratification and reduces unnecessary biopsies in men with suspected prostate cancer. *Cancers* **17**, 2257 (2025). 40647554 10.3390/cancers17132257 PMC12248439

[23] Parekh, S. et al. The Mount Sinai prebiopsy risk calculator for predicting any prostate cancer and clinically significant prostate cancer: development of a risk predictive tool and validation with advanced neural networking, prostate magnetic resonance imaging outcome database, and European randomized study of screening for prostate cancer risk calculator. *Eur. Urol. open Sci.* **41**, 45–54 (2022). 35813258 10.1016/j.euros.2022.04.017 PMC9257660

[24] Sungur, M., Aykaç, A., Aydin, M. E., Celik, O. & Kaya, C. Machine learning-based prediction of prostate biopsy necessity using psa, MRI, and hematologic parameters. *J. Clin. Med.* **14**, 183 (2024). 39797267 10.3390/jcm14010183 PMC11721894

[25] Rodrigues, A. C. et al. Improving clinically significant prostate cancer detection with a multimodal machine learning approach: a large-scale multicenter study. *Radiol. Imaging Cancer* **7**, e240507 (2025). 40815224 10.1148/rycan.240507 PMC12492419

[26] Kasneci, E. et al. ChatGPT for good? on opportunities and challenges of large language models for education. *Learn. Individ. Differ.* **103**, 102274 (2023).

[27] Liang, Z. et al. A survey of multimodel large language models. In *Proceedings of the 3rd International Conference on Computer, Artificial Intelligence and Control Engineering*, 405–409 (2024).

[28] Nguyen, D.-K. & Okatani, T. Improved fusion of visual and language representations by dense symmetric co-attention for visual question answering. In *Proceedings of the IEEE conference on computer vision and pattern recognition*, 6087–6096 (2018).

[29] Dou, Z.-Y. et al. Coarse-to-fine vision-language pre-training with fusion in the backbone. *Adv. Neural Inf. Process. Syst.* **35**, 32942–32956 (2022).

[30] Huang, J. et al. Clover: Towards a unified video-language alignment and fusion model. In *Proceedings of the IEEE/CVF Conference on Computer Vision and Pattern Recognition*, 14856–14866 (2023).

[31] Wang, F., Zhou, Y., Wang, S., Vardhanabhuti, V. & Yu, L. Multi-granularity cross-modal alignment for generalized medical visual representation learning. *Adv. Neural Inf. Process. Syst.* **35**, 33536–33549 (2022).

[32] Loshchilov, I. & Hutter, F. Decoupled weight decay regularization. *International Conference on Learning Representations* (2017).

