# Supplementary material for: Integrating multimodal clinical data with a large model for prostate cancer diagnosis
Source: NPJ Digit Med. 2026 Apr 25;9:497. doi: 10.1038/s41746-026-02670-x (PMC13324722; doi:10.1038/s41746-026-02670-x)
Supplement: Supplementary file 1 — Supplementary Information [file 41746_2026_2670_MOESM1_ESM.pdf]

Supplementary Material for:

Integrating Multimodal Clinical Data with a Large Model for Prostate Cancer Diagnosis

Table of Contents

- **Supplementary Table 1:** Multi-center cohort characteristics.
- **Supplementary Figure 1:** Performance evaluation using 5-fold cross-validation on the internal dataset.
- **Supplementary Figure 2:** Confusion Matrix Analysis between Traditional ML and Prost-LM.
- **Supplementary Note 1:** Logs of Model Interactions (QA Examples).

Supplementary Table 1: Multi-center cohort characteristics for prostate cancer diagnosis.

		Demographics		PSA		Pathology findings			
Center	ALL	Age (years)	BMI (kg/m2)	tPSA (ng/mL)	fPSA (ng/mL)	PCa (%)	Benign (%)	csPCa (%)	non-csPCa (%)
SNPH	1384	69 (10.1)	23.0 (21.3-25.1)	7.6 (3.9-14.7)	1.3 (0.6-2.5)	161 (11.6%)	1223 (88.4%)	125 (9%)	36 (2.6%)
CH	795	69 (7.7)	24.2 (22.4-26.1)	14.6 (8.5-32.3)	1.7 (1.0-3.4)	731 (91.9%)	64 (8.1%)	662 (83.3%)	69 (8.7%)
FHSU	546	70 (6.3)	23.8 (22.1-25.9)	15.3 (9.2-34.3)	1.9 (1.5-3.8)	546 (100.0%)	0 (0.0%)	531 (97.3%)	15 (2.7%)
YCFH	41	70 (8.0)	23.7 (21.9-25.6)	17.7 (9.6-33.7)	2.1 (1.3-4.4)	41 (100.0%)	0 (0.0%)	36 (87.8%)	5 (12.2%)
FHYL	479	72 (8.1)	22.8 (20.7-25.3)	19.8 (10.8-65.4)	2.7 (1.4-7.6)	218 (45.5%)	261 (54.5%)	200 (41.8%)	18 (3.8%)
FHZU	93	70 (9.9)	24.2 (22.3-26.4)	11.5 (5.7-34.7)	1.7 (0.8-4.1)	38 (40.9%)	55 (59.1%)	36 (38.7%)	2 (2.2%)
AHJU	602	71 (8.3)	23.4 (21.1-25.4)	10.7 (6.5-22.1)	2.0 (1.1-3.9)	271 (45.0%)	331 (55.0%)	201 (33.4%)	70 (11.6%)
Total	3940	70 (8.7)	23.9 (21.7-25.8)	12.8 (7.1-28.5)	1.8 (1.0-3.8)	2006 (50.9%)	1934 (49.1%)	1791 (45.5%)	215 (5.5%)

Note: Age is expressed as mean (SD); BMI, PSA are expressed as median (interquartile range). Abbreviations: BMI: body mass index; fPSA: free Prostate-Specific Antigen; tPSA: total Prostate-Specific Antigen; PCa: prostate cancer; Benign: benign prostatic disease; csPCa: clinically significant prostate cancer; non-csPCa: non-clinically significant prostate cancer.

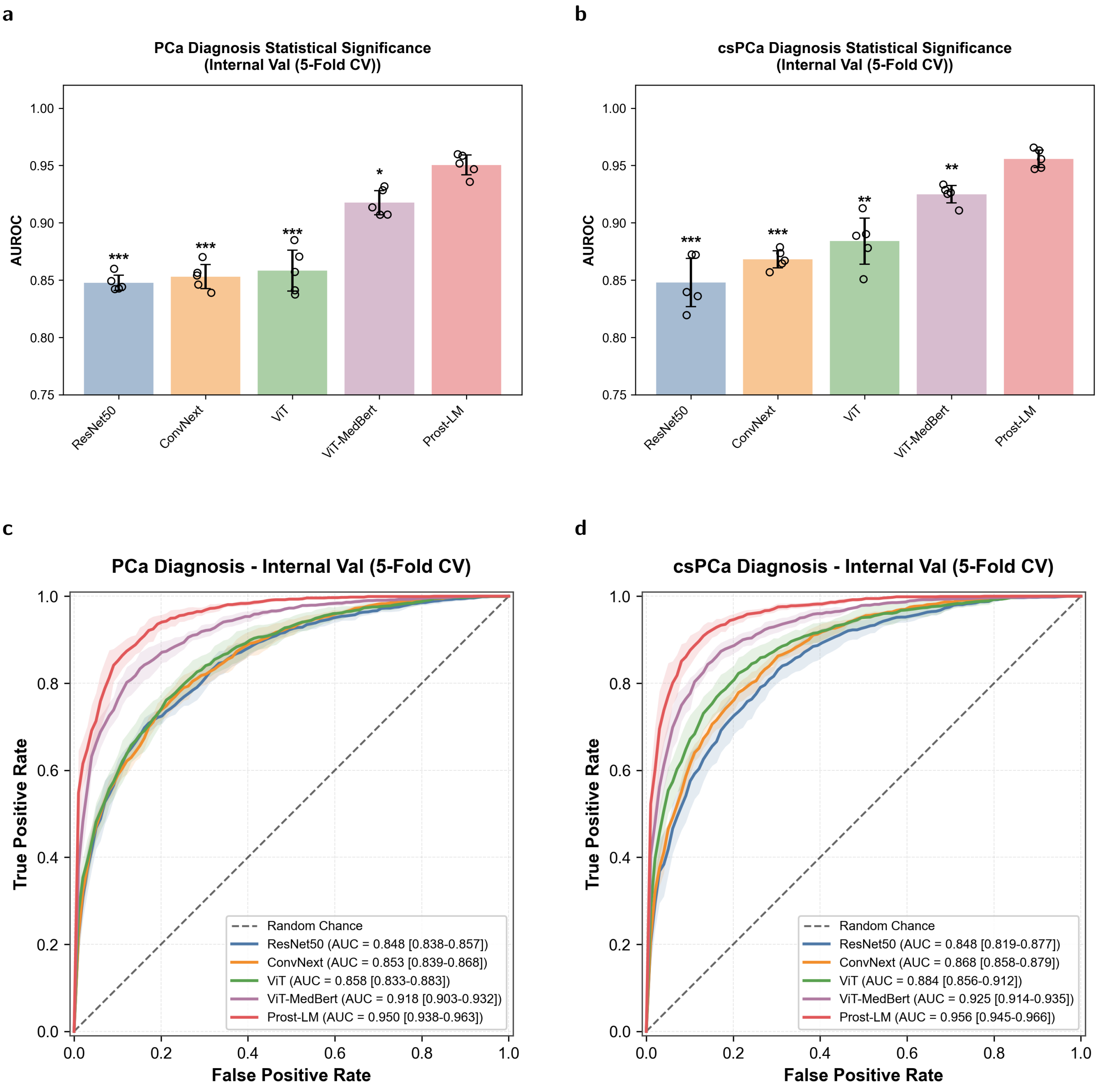

Supplementary Figure 1: **Performance evaluation using 5-fold cross-validation on the internal dataset.** **(a, b)** Bar charts showing the mean AUROC with standard deviation error bars for **(a)** PCa diagnosis and **(b)** csPCa diagnosis across 5 folds. Individual data points represent the result of each fold. Statistical significance was calculated using paired t-tests comparing Prost-LM against other methods (* $P < 0.05$, ** $P < 0.01$, *** $P < 0.001$).**(c, d)** Receiver Operating Characteristic (ROC) curves averaged over the 5 folds for **(c)** PCa diagnosis and **(d)** csPCa diagnosis. The solid lines represent the mean ROC, and the shaded areas represent the 95% confidence intervals (CI), demonstrating the stability of Prost-LM compared to single-modal and other multi-modal baselines.

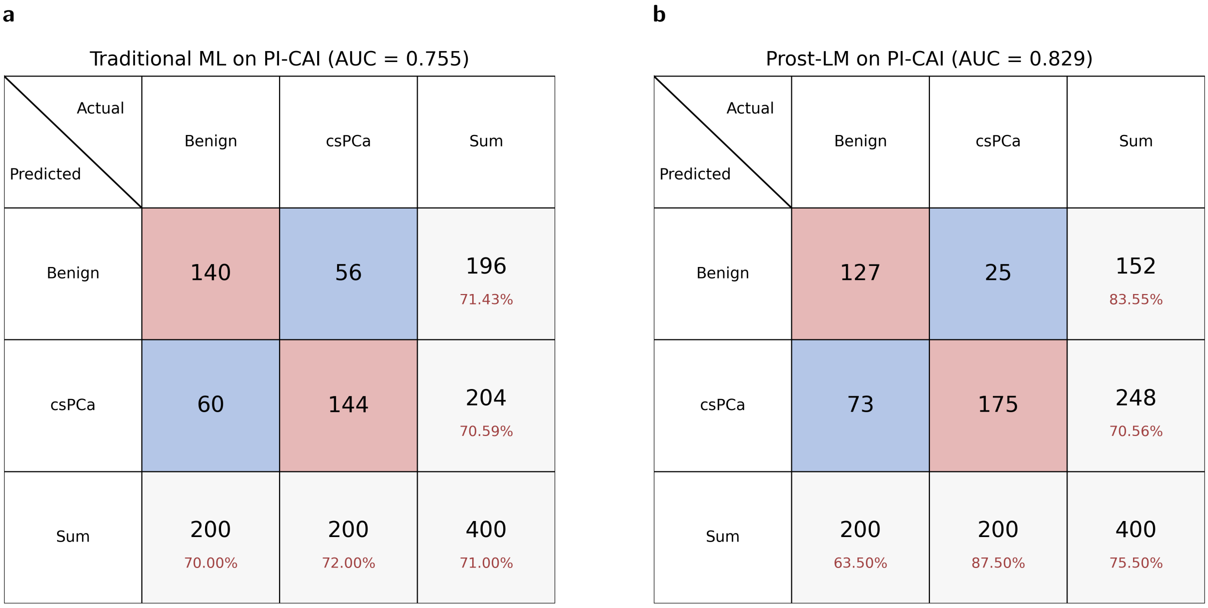

Supplementary Figure 2: **Confusion Matrix Analysis between Traditional ML and Prost-LM approaches.** Comparison of error distributions across (a) Traditional ML, and (b) Prost-LM. Prost-LM demonstrates a improved balance between sensitivity and specificity, significantly reducing the number of misclassified cases compared to the baselines, thereby validating its ability to model complex non-linear feature interactions.

Supplementary Note 1: Logs of Model Interactions

Below are detailed transcripts of the interactions between the clinical user and Prost-LM.

Case Study: Patient 1 (PI-RADS 3)

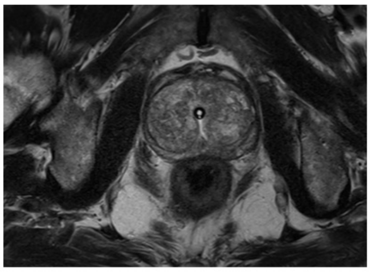

T2

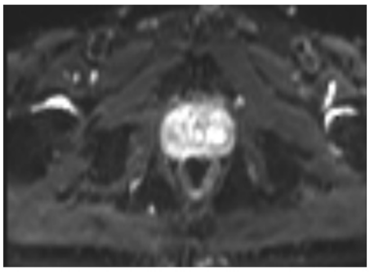

DWI

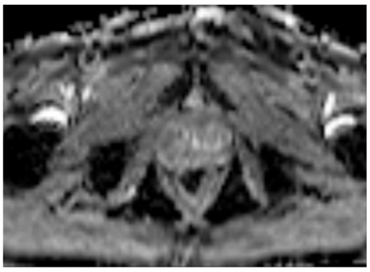

ADC

Demographics: Age: 78, Height: 167 cm, Weight: 65 kg
PSA Profile: tPSA: 8.73 ng/mL, fPSA: 1.76 ng/mL (Ratio ≈ 0.20)
Symptoms: Found elevated PSA 1 week ago.
History: Aortic stent placement 2 years ago for aortic dissection intramural hematoma; self-reported good recovery. History of hypertension (max 180/100 mmHg), medication irregular.

User

Is PSA abnormal or elevated in this case?

Model

The total PSA (tPSA) is elevated at 8.73 ng/mL. The free PSA (fPSA) is 1.76 ng/mL, with an f/tPSA ratio of approximately 0.20.

User

What do the MRI images suggest?

Model

On T2WI, the prostate is enlarged, dominated by nodular and heterogeneous signal changes in the central gland (transition zone), with a compressed and thinned peripheral zone. DWI does not show a definite focal marked hyperintensity, and ADC does not show a corresponding clear focal low signal, overall lacking a typical markedly diffusion-restricted suspicious lesion. Dynamic contrast-enhanced images are not provided, so enhancement cannot be assessed.

User

Based on the available clinical text information and MRI images, what is the most likely diagnosis?

Model

The imaging pattern is mainly nodular transition-zone enlargement without a definite focal diffusion-restricted lesion, which is more suggestive of benign prostatic hyperplasia-related changes. Given the elevated PSA, fluctuation related to benign hyperplasia/inflammation is possible, but the provided images do not strongly support a clear diagnosis of prostate cancer.

User

If this is a benign condition, which specific benign disease is most likely?

Model

The most likely benign condition is benign prostatic hyperplasia (BPH), i.e., nodular/transition-zone hyperplasia.

Case Study: Patient 2 (PI-RADS 3)

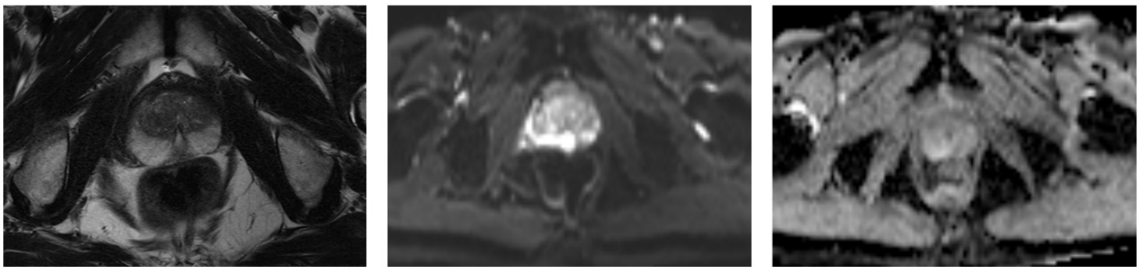

Demographics: Age: 60, Height: 167 cm, Weight: 71 kg
PSA Profile: tPSA: 1.09 ng/mL, fPSA: 0.24 ng/mL (Ratio $\approx$ 0.22)
Symptoms: Difficulty in urination for more than half a year.
History: Diseases and infectious diseases: Slightly high blood pressure, history of chronic bronchitis, and history of lacunar infarction. Surgical and trauma history: Radiofrequency ablation was performed for atrial fibrillation half a year ago. Had surgery for lower extremity venous thrombosis. Clopidogrel and aspirin have been discontinued.

User

Is PSA abnormal or elevated in this case?

Model

The total PSA (tPSA) is 1.09 ng/mL, which is not elevated and is within commonly used reference ranges. The free PSA (fPSA) is 0.24 ng/mL, with an f/tPSA ratio of approximately 0.22.

User

What do the MRI images suggest?

Model

On T2WI, the prostate appears mildly enlarged, with heterogeneous/nodular changes in the central gland (transition zone), and no definite focal abnormal low-signal lesion in the peripheral zone. DWI shows no clear focal marked hyperintensity, and ADC shows no corresponding definite focal hypointensity, overall lacking a typical markedly diffusion-restricted lesion. Dynamic contrast-enhanced images are not provided, so enhancement cannot be assessed.

User

Based on the available clinical text information and MRI images, what is the most likely diagnosis?

Model

Given the non-elevated PSA and an imaging pattern dominated by transition-zone hyperplastic/nodular changes without a definite focal diffusion-restricted lesion, a benign process is most likely. Overall, the findings are most consistent with benign prostatic hyperplasia-related changes.

User

If this is a benign condition, which specific benign disease is most likely?

Model

The most likely benign condition is benign prostatic hyperplasia (BPH), i.e., nodular/transition-zone hyperplasia.

Case Study: Patient 3 (PI-RADS 3)

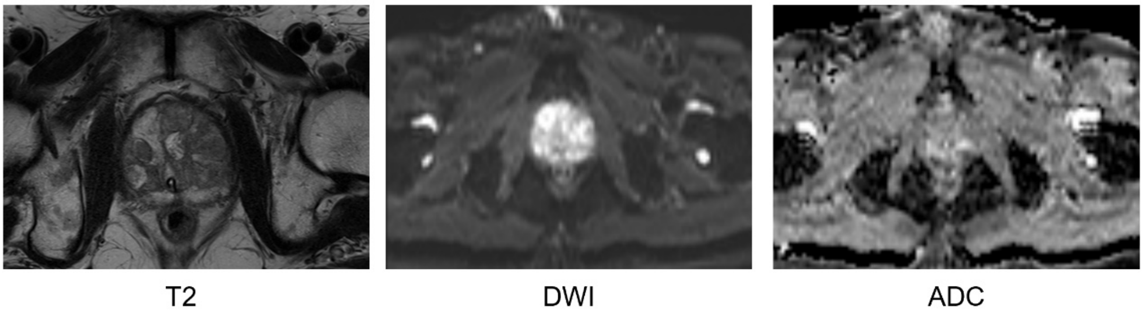

Demographics: Age: 80, Height: 170 cm, Weight: 67.5 kg
PSA Profile: tPSA: 21.6 ng/mL, fPSA: 6.18 ng/mL (Ratio ≈ 0.29)
Symptoms: Urinary retention for 1 day.
History: Visited local clinic for prostatitis 1 month ago; took Qianlieshutong capsules and Amoxicillin

with poor effect.

User

Is PSA abnormal or elevated in this case?

Model

The total PSA (tPSA) is markedly elevated at 21.6 ng/mL. The free PSA (fPSA) is 6.18 ng/mL, with an f/tPSA ratio of approximately 0.29.

User

What do the MRI images suggest?

Model

On T2WI, the prostate is enlarged with heterogeneous/nodular changes in the central gland (transition zone), and the peripheral zone appears compressed/thinned. DWI shows no clear focal marked hyperintensity, and ADC shows no corresponding definite focal low signal, overall lacking a typical markedly diffusion-restricted highly suspicious lesion. Dynamic contrast-enhanced images are not provided, so enhancement cannot be assessed.

User

Based on the available clinical text information and MRI images, what is the most likely diagnosis?

Model

The imaging pattern is dominated by transition-zone nodular hyperplasia without a definite focal diffusion-restricted lesion, which favors a benign process. Given acute urinary retention, a recent history of prostatitis, and markedly elevated PSA, the overall picture is more consistent with benign prostatic hyperplasia with active/inflamed changes causing PSA fluctuation.

User

If this is a benign condition, which specific benign disease is most likely?

Model

The most likely benign condition is benign prostatic hyperplasia (BPH; nodular transition-zone hyperplasia), possibly with concurrent active prostatitis.

Case Study: Patient 4 (PI-RADS 3)

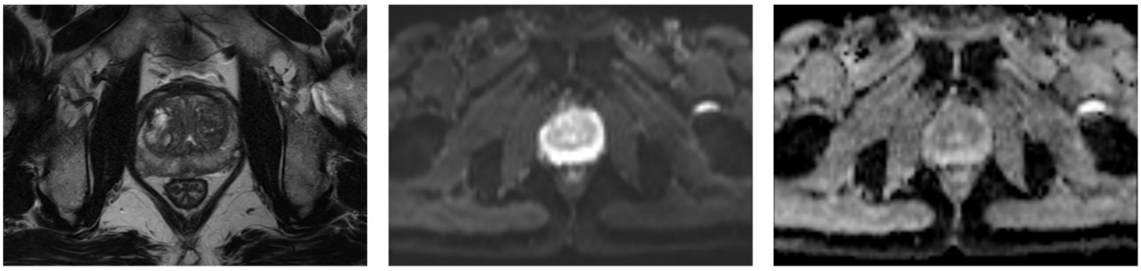

T2 DWI ADC

Demographics: Age: 60, Height: 169 cm, Weight: 66 kg
PSA Profile: tPSA: 12.1 ng/mL, fPSA: 2.05 ng/mL (Ratio ≈ 0.17)
Symptoms: Known prostatic cystic hyperplasia for > 20 days.
History: Renal cyst surgery 26 years ago; right mandibular cyst surgery 3 years ago; thyroid nodule surgery 2 years ago. Diabetes for > 5 years (on Metformin, Acarbose, well controlled). History of bronchitis.

User

Is PSA abnormal or elevated in this case?

Model

The total PSA (tPSA) is elevated at 12.1 ng/mL. The free PSA (fPSA) is 2.05 ng/mL, with an f/tPSA ratio of approximately 0.17.

User

What do the MRI images suggest?

Model

On T2WI, the prostate appears enlarged with nodular/heterogeneous signal changes in the central gland (transition zone), and the peripheral zone is relatively compressed/thinned. DWI shows no clear focal marked hyperintensity, and ADC shows no corresponding definite focal hypointensity, overall lacking a typical markedly diffusion-restricted highly suspicious lesion. Dynamic contrast-enhanced images are not provided, so enhancement cannot be assessed.

User

Based on the available clinical text information and MRI images, what is the most likely diagnosis?

Model

The imaging pattern is dominated by transition-zone nodular hyperplasia without a definite focal diffusion-restricted lesion, which overall favors a benign process. With an elevated PSA, benign hyperplasia with concurrent inflammation/irritation causing PSA fluctuation is more likely.

User

If this is a benign condition, which specific benign disease is most likely?

Model

The most likely benign condition is benign prostatic hyperplasia (BPH; nodular transition-zone/central-gland hyperplasia), possibly with concurrent active prostatitis.

Case Study: Patient 5 (PI-RADS 3)

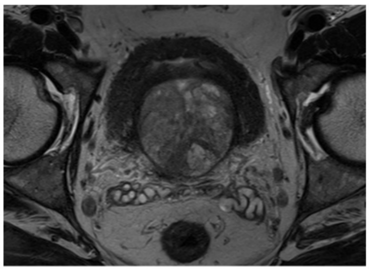

T2

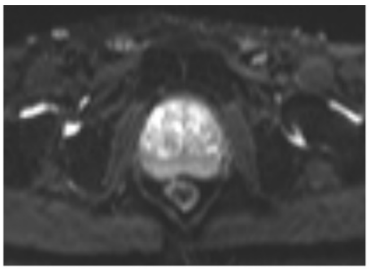

DWI

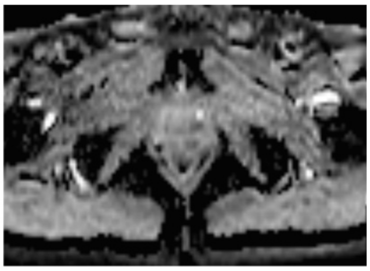

ADC

Demographics: Age: 76, Height: 170 cm, Weight: 70 kg
PSA Profile: tPSA: 10.3 ng/mL, fPSA: 1.68 ng/mL (Ratio ≈ 0.16)
Symptoms: Difficulty urinating for 2 years, aggravated for 20 days.
History: Hypertension for 5 years (intermittent medication, poor control); cerebral infarction 26 years ago; Parkinson's syndrome for 1 year. Denies diabetes, hepatitis, or tuberculosis.

User

Is PSA abnormal or elevated in this case?

Model

The total PSA (tPSA) is elevated at 10.3 ng/mL. The free PSA (fPSA) is 1.68 ng/mL, with an f/tPSA ratio of approximately 0.16.

User

What do the MRI images suggest?

Model

On T2WI, the prostate is enlarged, dominated by nodular/heterogeneous signal changes in the central gland (transition zone), with a relatively thinned peripheral zone. DWI does not show a definite single focal marked hyperintense lesion, and ADC does not show a corresponding clear focal low-signal area, overall lacking a typical markedly diffusion-restricted highly suspicious lesion. Dynamic contrast-enhanced images are not provided, so enhancement cannot be assessed.

User

Based on the available clinical text information and MRI images, what is the most likely diagnosis?

Model

Given that the imaging pattern is mainly transition-zone hyperplasia without a definite focal diffusion-restricted lesion, a benign process is favored. With worsening urinary symptoms and an elevated PSA, benign prostatic hyperplasia with inflammation/irritation causing PSA fluctuation is more likely.

User

If this is a benign condition, which specific benign disease is most likely?

Model

The most likely benign condition is benign prostatic hyperplasia (BPH; nodular/transition-zone hyperplasia), possibly with concurrent active prostatitis.

Case Study: Patient 6 (PI-RADS 3-4)

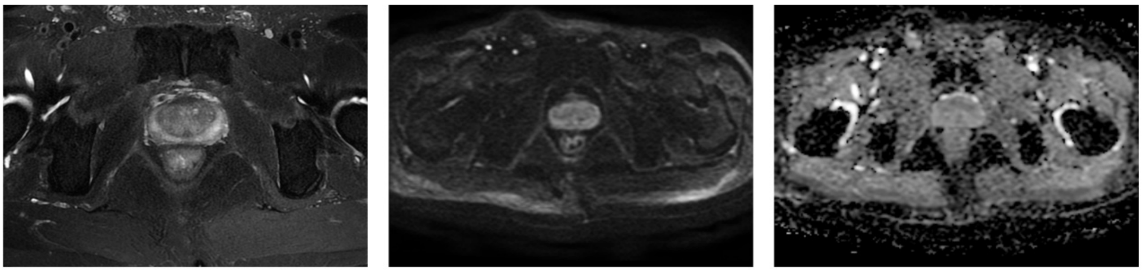

T2 DWI ADC

Demographics: Age: 52, Height: 166 cm, Weight: 67 kg
PSA Profile: tPSA: 8.278 ng/mL, fPSA: 0.421 ng/mL (Ratio ≈ 0.05)
Symptoms: Fever with dysuria for 4 days (accompanied by frequency, urgency, and gross hematuria).
History: Onset of fever (max 38.0°C) 4 days ago. Outside urinalysis suggested UTI; ultrasound showed BPH with calcification. Currently on IV Levofloxacin and oral Fosfomycin. History of smoking and alcohol use (30+ years).

User

Is PSA abnormal or elevated in this case?

Model

The total PSA (tPSA) is elevated at 8.278 ng/mL. The free PSA (fPSA) is 0.421 ng/mL, with an f/tPSA ratio of approximately 0.05.

User

What do the MRI images suggest?

Model

On T2WI, there is no obvious large mass, but a suspicious focal low-signal lesion is suggested in the right peripheral zone. DWI shows corresponding focal hyperintensity with relatively low signal on ADC, indicating focal diffusion restriction. Dynamic contrast-enhanced images are not provided, so enhancement cannot be assessed.

User

Based on the available clinical text information and MRI images, what is the most likely diagnosis?

Model

Given the acute infectious presentation (fever, dysuria, frequency/urgency, and gross hematuria), the focal diffusion-restricted lesion is more likely to represent inflammation-related change (focal prostatitis). However, a small focus of prostate cancer remains in the differential; PSA follow-up after antibiotics and repeat MRI if needed are recommended.

User

If this is a benign condition, which specific benign disease is most likely?

Model

The most likely benign condition is acute (or focal) bacterial prostatitis / UTI-associated inflammatory change in the prostate.

Case Study: Patient 7 (PI-RADS 3)

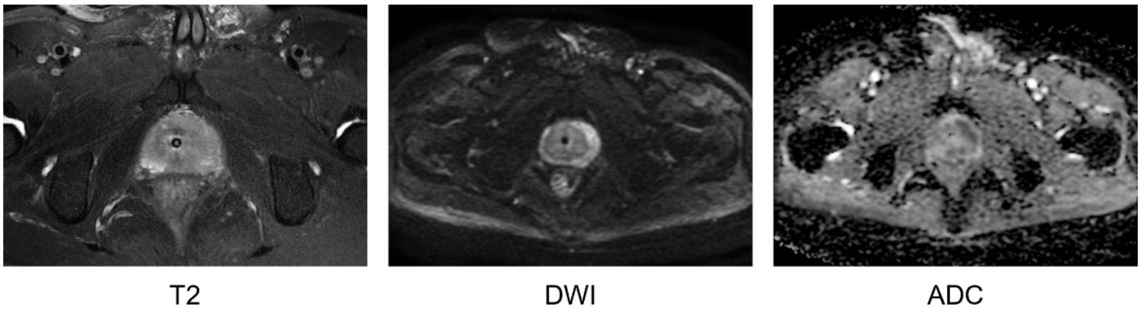

Demographics: Age: 68, Height: 177 cm, Weight: 65 kg
PSA Profile: tPSA: 61.059 ng/mL, fPSA: 4.997 ng/mL (Ratio ≈ 0.08)
Symptoms: Difficulty urinating for 10 days, fever for > 72 hours.
History: Urinary difficulty and fever 10 days ago requiring catheterization; symptoms recurred with high fever (39.0°C) after catheter removal. Emergency re-catheterization performed. Fever persisted despite 3 days of IV Levofloxacin. Current medication: Biktarvy daily.

User

Is PSA abnormal or elevated in this case?

Model

The total PSA (tPSA) is markedly elevated at 61.059 ng/mL. The free PSA (fPSA) is 4.997 ng/mL, with an f/tPSA ratio of approximately 0.08.

User

What do the MRI images suggest?

Model

On T2WI, the prostate appears enlarged with overall heterogeneous signal, and the peripheral zone shows relatively diffuse changes rather than a single well-defined nodule. DWI does not show a definite isolated focal markedly hyperintense mass-like lesion, and ADC does not show a corresponding clear focal marked low signal; the pattern looks more diffuse/patchy rather than a typical solitary tumor focus. Dynamic contrast-enhanced images are not provided, so enhancement cannot be assessed.

User

Based on the available clinical text information and MRI images, what is the most likely diagnosis?

Model

Given the acute infectious context (fever, urinary difficulty, catheterization history) and an imaging pattern dominated by diffuse changes without a clear tumor-like focal lesion, the most likely diagnosis is acute prostatitis (which can cause marked PSA elevation). PSA should be rechecked after antibiotics and MRI can be repeated if needed to exclude any residual suspicious focus.

User

If this is a benign condition, which specific benign disease is most likely?

Model

The most likely benign condition is acute bacterial prostatitis with diffuse inflammatory change.

Case Study: Patient 8 (PI-RADS 4)

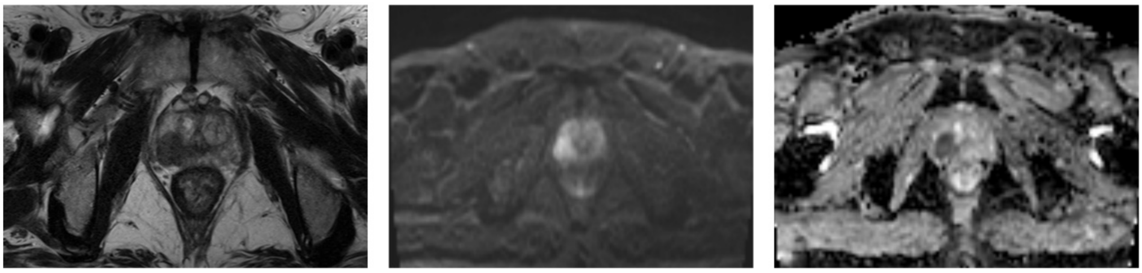

Demographics: Age: 59, Height: 172 cm, Weight: 75 kg
PSA Profile: tPSA: 17.0 ng/mL, fPSA: 3.04 ng/mL (Ratio $\approx$ 0.18)
Symptoms: Hematuria for > 1 day.
History: Diabetes > 10 years (Metformin, Dulaglutide); Hypertension > 10 years (Sacubitril/Valsartan, Nifedipine); Old myocardial infarction noted in 2020; Hepatitis B; Hemorrhoid surgery in 2012.

User

Is PSA abnormal or elevated in this case?

Model

The total PSA (tPSA) is elevated at 17.0 ng/mL. The free PSA (fPSA) is 3.04 ng/mL, with an f/tPSA ratio of approximately 0.18.

User

What do the MRI images suggest?

Model

On T2WI, there is a suspicious focal low-signal lesion in the right peripheral zone. DWI shows corresponding focal hyperintensity with relatively low signal on ADC, indicating focal diffusion restriction. Dynamic contrast-enhanced images are not provided here, so enhancement cannot be assessed.

User

Based on the available clinical text information and MRI images, what is the most likely diagnosis?

Model

Given the elevated PSA and a focal diffusion-restricted lesion in the peripheral zone, the most likely diagnosis is prostate cancer (peripheral-zone lesion). Inflammation can mimic this, but this lesion appears more tumor-like; targeted/systematic biopsy is recommended for confirmation.

User

If this is a tumor, is it likely to be clinically significant prostate cancer (csPCa)?

Model

The lesion shows a typical peripheral-zone diffusion-restriction pattern, suggesting a higher likelihood of csPCa. Definitive csPCa classification still requires biopsy-based grading and assessment of tumor burden.

Case Study: Patient 9 (PI-RADS 5)

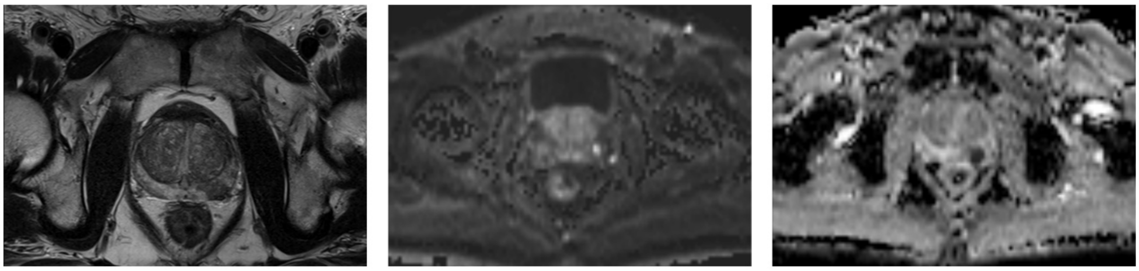

T2 DWI ADC

Demographics: Age: 65, Height: 167 cm, Weight: 63 kg
PSA Profile: tPSA: 57.4 ng/mL, fPSA: 2.36 ng/mL (Ratio $\approx$ 0.04)
Symptoms: Found elevated PSA 2 months ago.
History: Diabetes $>$ 10 years (Insulin, well controlled); History of surgery for cardia lesion; History of heart disease. Denies cerebrovascular disease, hepatitis, tuberculosis, or malaria.

User

Is PSA abnormal or elevated in this case?

Model

The total PSA (tPSA) is markedly elevated at 57.4 ng/mL. The free PSA (fPSA) is 2.36 ng/mL, with an f/tPSA ratio of approximately 0.04.

User

What do the MRI images suggest?

Model

On T2WI, there is a focal low-signal lesion in one side of the peripheral zone. DWI shows corresponding focal hyperintensity with relatively low signal on ADC, indicating marked diffusion restriction. Dynamic contrast-enhanced images are not provided here, so enhancement cannot be assessed.

User

Based on the available clinical text information and MRI images, what is the most likely diagnosis?

Model

Given the markedly elevated PSA and a focal diffusion-restricted peripheral-zone lesion, the most likely diagnosis is prostate cancer. Pathologic confirmation with targeted plus systematic biopsy is recommended.

User

If this is a tumor, is it likely to be clinically significant prostate cancer (csPCa)?

Model

The lesion shows a typical peripheral-zone pattern of marked diffusion restriction, suggesting a higher likelihood of csPCa. Definitive csPCa classification still requires biopsy-based Gleason grading and assessment of tumor burden.

Case Study: Patient 10 (PI-RADS 4)

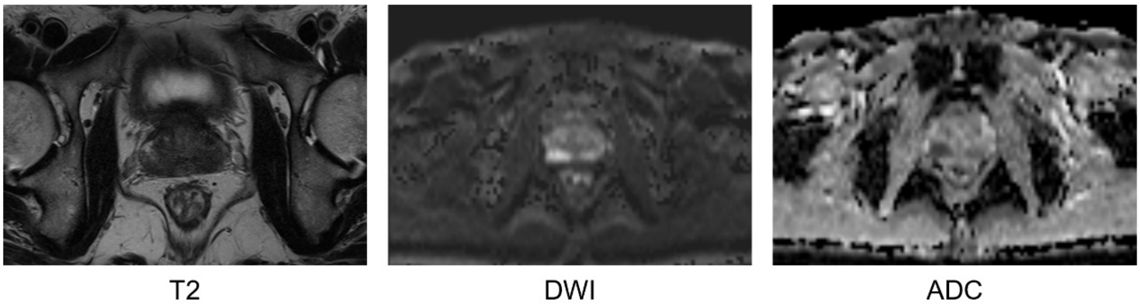

Demographics: Age: 70, Height: 170 cm, Weight: 70 kg
PSA Profile: tPSA: 12.2 ng/mL, fPSA: 0.75 ng/mL (Ratio $\approx$ 0.06)
Symptoms: Lower abdominal distension and pain for $>$ 1 year.
History: History of trauma surgery (hand debridement/suture, right ankle fracture surgery); Fatty liver for $>$ 7 years (untreated). Denies hypertension, heart disease, diabetes, or cerebrovascular disease.

User

Is PSA abnormal or elevated in this case?

Model

The total PSA (tPSA) is elevated at 12.2 ng/mL. The free PSA (fPSA) is 0.75 ng/mL, with an f/tPSA ratio of approximately 0.06, which is relatively low.

User

What do the MRI images suggest?

Model

On T2WI, the prostate is mildly enlarged with heterogeneous signal in the central gland, and the peripheral zone shows a suspicious patchy low-signal area. DWI demonstrates focal/patchy hyperintensity in the peripheral zone (more evident on the right), with corresponding low signal on ADC, indicating diffusion restriction. Dynamic contrast-enhanced images are not provided here, so enhancement cannot be assessed.

User

Based on the available clinical text information and MRI images, what is the most likely diagnosis?

Model

Given the elevated PSA and diffusion-restricted abnormality in the peripheral zone, the most likely diagnosis is prostate cancer (with a dominant suspicious lesion in the right peripheral zone). Targeted plus systematic biopsy is recommended for pathologic confirmation.

User

If this is a tumor, is it likely to be clinically significant prostate cancer (csPCa)?

Model

The peripheral-zone diffusion restriction is fairly clear, suggesting a higher likelihood of csPCa. Definitive csPCa classification still requires biopsy-based Gleason grading and assessment of tumor burden.
